# Horizontal proton transfer across the antiporter-like subunits in mitochondrial respiratory complex I†

**DOI:** 10.1039/d3sc01427d

**Published:** 2023-05-10

**Authors:** Oleksii Zdorevskyi, Amina Djurabekova, Jonathan Lasham, Vivek Sharma

**Affiliations:** a Department of Physics, University of Helsinki Helsinki Finland vivek.sharma@helsinki.fi; b HiLIFE Institute of Biotechnology, University of Helsinki Helsinki Finland

## Abstract

Respiratory complex I is a redox-driven proton pump contributing to about 40% of total proton motive force required for mitochondrial ATP generation. Recent high-resolution cryo-EM structural data revealed the positions of several water molecules in the membrane domain of the large enzyme complex. However, it remains unclear how protons flow in the membrane-bound antiporter-like subunits of complex I. Here, we performed multiscale computer simulations on high-resolution structural data to model explicit proton transfer processes in the ND2 subunit of complex I. Our results show protons can travel the entire width of antiporter-like subunits, including at the subunit–subunit interface, parallel to the membrane. We identify a previously unrecognized role of conserved tyrosine residues in catalyzing horizontal proton transfer, and that long-range electrostatic effects assist in reducing energetic barriers of proton transfer dynamics. Results from our simulations warrant a revision in several prevailing proton pumping models of respiratory complex I.

## Abstract 2

Proton transfer in the membrane domain of complex I.

## Graphical abstract

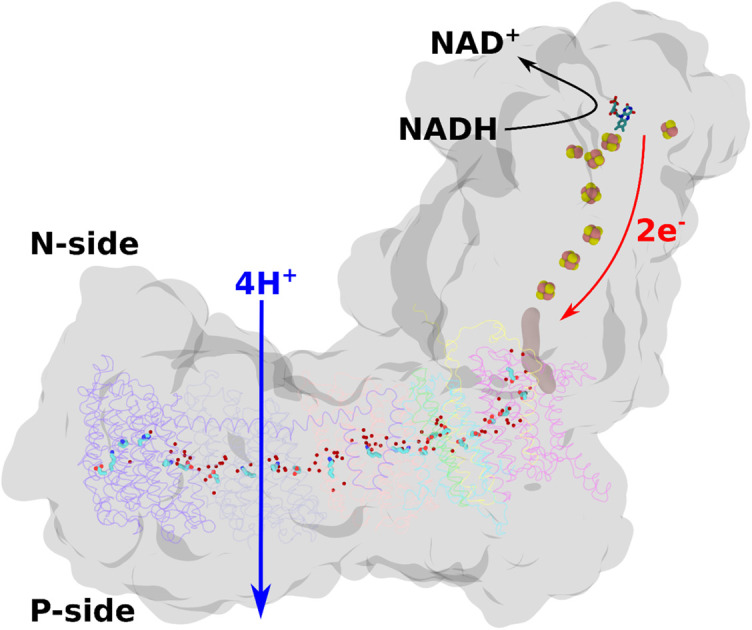

## Introduction

Mitochondrial respiratory complex I (NADH:ubiquinone oxidoreductase) is a large (∼1 MDa) enzyme in the electron transport chain.^1,2^ It couples the processes of NADH oxidation and quinone reduction to the translocation of four protons across the inner mitochondrial membrane. The generated protonmotive force is utilized by the respiratory chain for ATP synthesis. Despite a plethora of high-resolution structures of complex I,^3–10^ its proton pumping mechanism still remains a mystery.

**Fig. 1:**
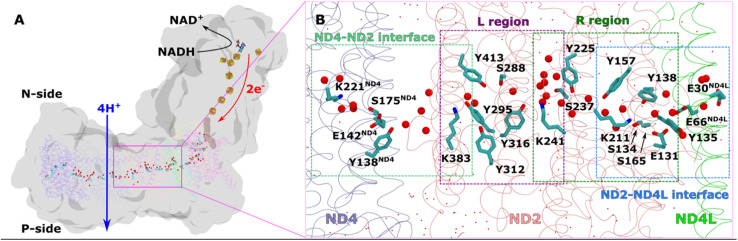
Overall architecture of respiratory complex I and its membrane bound antiporter-like subunit (A) structure of mitochondrial complex I from *Y. lipolytica* (PDB 7O71). The core subunits of the membrane arm (ND1, ND2, ND3, ND4, ND5, ND4L, ND6) are shown in the ribbons representation, whereas rest of the protein as grey surface. Structurally-resolved water molecules (red spheres) and conserved residues (cyan) in the central hydrophilic axis, which connects the Q tunnel (brown surface) with terminal end of membrane domain, are shown. Protein-bound electron transfer components FeS clusters (yellow-orange spheres) and FMN molecule (licorice) are also displayed. Proton and electron transfer are marked in blue and red arrows, respectively. (B) Colored dotted boxes highlight the QM regions (as part of QM/MM free energy simulations) studied in the work. Purple (L region), green (R region), light blue (ND2–ND4L interface) and light green (ND4–ND2 interface). The “big QM region” comprises the protein residues and water molecules from both R and L regions together. All amino acid residues that are part of each of the QM regions are displayed in licorice, and structural waters as red spheres. Water molecules that are not part of QM, but MM, are shown in dotted representation (red).

Mitochondrial complex I comprises 14 core subunits, organized into a characteristic L-shape: a peripheral arm where redox reactions take place, and a membrane part working as a proton pump (Fig. 1A). The core subunits are shared between bacterial and mitochondrial enzymes. However, the latter have several supernumerary (accessory) subunits, the function of which largely remains unknown. In the core membrane domain of complex I, the three antiporter-like subunits (ND2, ND4 and ND5, Fig. 1A) are responsible for transferring protons from the N side to the P side of the membrane.^1,2^ Interestingly, these three antiporter-like subunits are interlinked to four other membrane-bound core subunits (ND6, ND4L, ND3 and ND1) by a central hydrophilic axis (Fig. 1A). This ‘river’ of charged and polar residues together with water molecules provide connectivity between the quinone binding tunnel and the terminal end of membrane arm, spanning *ca.* 200 Å (Fig. 1A). The putative proton transfer pathways in antiporter-like subunits have been investigated by structural biology techniques, site-directed mutagenesis and computational approaches.^3–16^ It has been proposed that each of the three antiporter-like subunits pick protons from the N side and release to the P side as a response to redox activity in the quinone binding tunnel. Proton transfer from the N side is assumed to first occur to a conserved Lys residue in transmembrane helix (TMH) 8 (Lys241 in ND2), located in the central part of the subunit (all amino acid numbering corresponds to complex I from *Yarrowia lipolytica*, unless otherwise stated). Subsequent proton transfer from this to another conserved lysine in ND2 (Lys383 or a glutamate in ND4 but with function shared^13^), and putatively to the P side is driven by the dynamics and/or protonation reactions of conserved Lys/Glu pair from TMH 7/5.^1,15,16^ However, how this is achieved at a molecular level remains elusive and debated.^6,15^

Recently, proton pumping models in which antiporter-like subunits act as self-containing proton pumps have been challenged by the high-resolution structural data on complex I.^6^ In contrast to a clear hydration observed towards the P side exit in terminal ND5 subunit,^10,14^ analogous path in ND2/4 subunits is blocked by bulky hydrophobic residues.^10^ This has led to the proposal that only the terminal antiporter-like subunit ND5 is responsible for the release of protons to the P side,^6,10^ whereas protons are fed into the central hydrophilic axis from the N side *via* pathways present in all three antiporter-like subunits.^10^

A recent high-resolution (2.4 Å) cryo-EM structure of *Y. lipolytica* complex I revealed extensive hydration in the middle of the ND2 subunit (Fig. 1B). Refinement with classical molecular dynamics (MD) simulations showed a fully hydrogen bonded connectivity across the antiporter-like subunit.^10^ This led to the suggestion that protons can move on the hydrogen-bonded proton wire, and can travel long distances in the membrane domain of complex I. Here, based on classical and quantum mechanical simulations, we show protons can indeed migrate through the hydrated path of antiporter-like subunits and crucially also at the subunit–subunit interface. To track the energetic feasibility of proton diffusion across the entire width of antiporter-like subunits, we performed quantum mechanical/molecular mechanical (QM/MM) free energy calculations, which reveal low energy barriers of proton transfer dynamics in the membrane domain. Our results suggest long range proton transport occurs in the membrane-bound subunits of complex I and justifies the revision of models of proton pumping in the membrane domain of respiratory complex I.

## Results

### Proton transfer pathways in the ND2 subunit studied by QM/MM MD simulations

We applied QM/MM free energy simulations on the high-resolution complex I structure from *Y. lipolytica*.^10^ We restricted the size of our QM/MM model system to 6 core subunits: ND1, ND2, ND3, ND4, ND4L, and ND6. The hydrated central axis of ND2 subunit was treated with full QM description and the rest of the system was described by MM framework (see Methods). To keep the computationally demanding free energy simulations tractable, we split the ND2 QM region into two parts (see Fig. 1B; S1A and B, ESI†). The first one (R) comprises the region between the highly-conserved Glu131 of conserved Lys/Glu pair from TMH7/5 and the central Lys241 (proximal to Lys/Glu pair, see Fig. 1). The second region (L region) spans the section between Lys241 and terminal Lys383, which is distal to the Lys/Glu pair and closer to ND2/ND4 interface (Fig. 1). These four highly conserved titratable residues (Lys211 and Glu131 of Lys/Glu pair, central Lys241 and terminal Lys383) are all known to be important for the proton pumping activity of complex I.^17,18^

Based on p*K*_a_ calculations^19^ and classical MD simulations, we first modelled these residues in their standard (charged) protonation states and performed unbiased QM/MM MD (molecular dynamics) simulations. We did not observe any spontaneous proton conductance in either of the two ND2 regions (R or L), or in a QM/MM MD simulation of a much larger QM region (Fig. 1B, see methods, Tables S1–S4†). We thus created proton vacancies to drive the forward proton transfer *via* a Grotthuss “hole” mechanism.^20^ A proton vacancy at the end of the “L” region was created by neutralizing terminal Lys383. p*K*_a_ calculations performed on snapshots obtained from classical simulations demonstrate low proton affinity of Lys383 (Fig. S2†), thereby justifying its modeling as a proton hole. A similar low proton affinity of a buried lysine residue has also been observed in another protein system.^21^ A ∼5.5 ps QM/MM MD trajectory did not result in any spontaneous proton transfer to neutral Lys383 from central Lys241 (protonated) suggesting the presence of energy barriers that could not be overcome in unbiased simulations. However, an excess proton (modelled as H_3_O^+^ ion) at several structurally-conserved water positions (Fig. S1A†), resulted in rapid proton transfer to terminal Lys383, within 50–200 fs of the unbiased QM/MM MD simulation runtime. Remarkably, this involved the deprotonation (and re-protonation) of highly conserved tyrosine (Tyr413 and Tyr316) and Ser288 residues in the ND2 subunit.

**Fig. 2:**
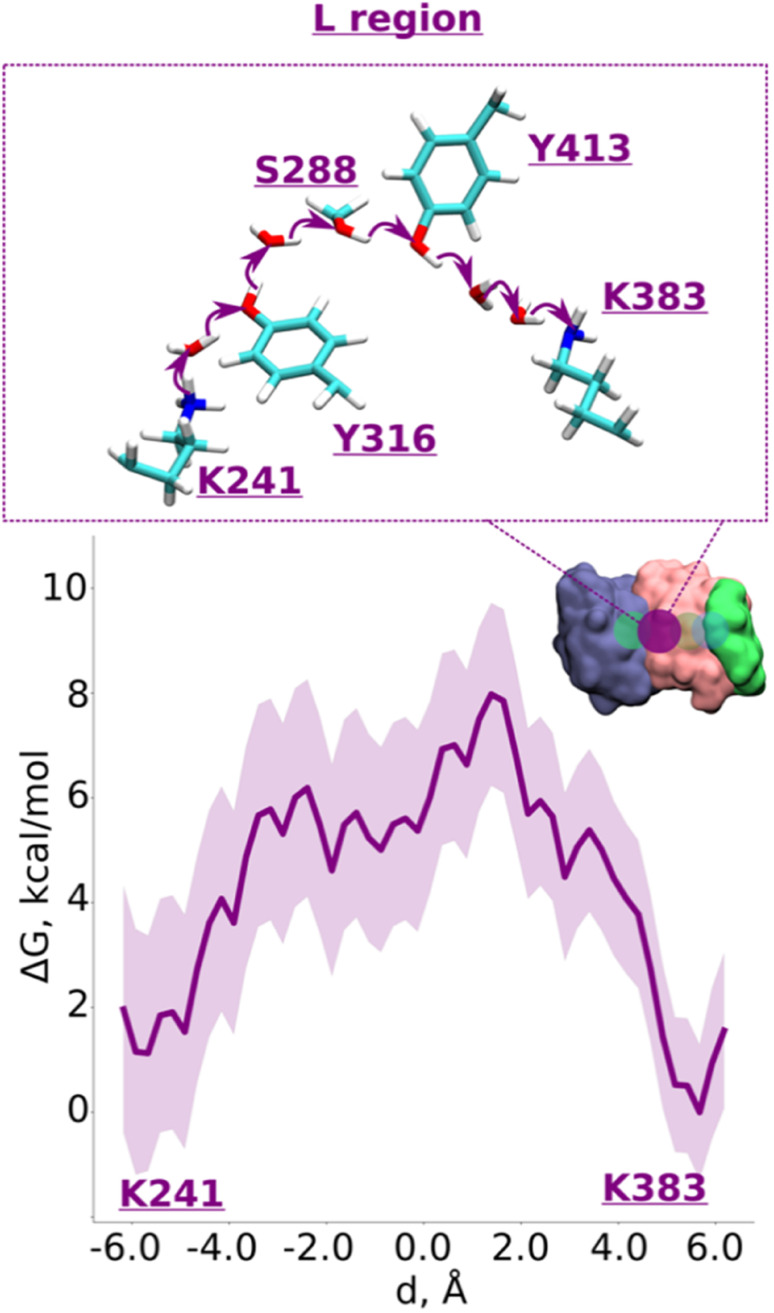
Proton transfer from central to terminal lysine (L region). Proton transfer occurs from Lys241 (protonated) → H_2_O → Tyr316 (neutral) → H_2_O → Ser288 (neutral) → Tyr413 (neutral) → H_2_O → H_2_O → Lys383 (neutral) as depicted in the top panel. The bottom panel shows PMF (potential of mean force) profile of proton transfer derived from our QM/MM umbrella sampling simulations. Purple shaded area around the bold purple line depicts the bootstrapping error range (see also methods). Occupational histograms for the reaction coordinates are shown in ESI, Fig. S17A.† The inset depicts the core membrane subunits of respiratory complex I: ND4 (dark blue), ND2 (pink), and ND4L (green). The purple circle denotes the QM region where the proton transfer is investigated.

With the indication that deprotonation of these residues may be responsible for the barrier in proton transfer dynamics, we initiated umbrella sampling (US) simulations to induce proton transfer to neutral Lys383 from the protonated Lys241. The potential of mean force (PMF) profile derived from this setup shows an energy barrier (∼6.5 kcal mol^−1^, Fig. 2, note the statistical error bars displayed in the figure) with a favorable proton position on Lys383. The obtained activation energy barrier is similar to the one obtained previously based on calculations on a lower resolution structure of mammalian complex I.^16^ The additional analysis of the hydrogen bonding patterns along the pathway suggests the preservation of the distinct hydrogen bond network in initial, final, and intermediate stages of proton transfer (ESI, Fig. S3 and S4†). Interestingly, in place of an asparagine (Asn292) in *Y. lipolytica* complex I, mammalian complex I consists of a titratable residue His186 (*Mus musculus* complex I numbering, ESI, Fig. S5†). Given that the energetics obtained in two studies are comparable, we suggest a titratable residue at this location is not necessary, instead, proton transfer would follow the path discussed here involving protonation/deprotonation of highly conserved Tyr413 (Fig. 2 and S1A, ESI†). Indeed, proton transfer energetics obtained on the path from Tyr413 to Lys383 revealed a similar barrier of 6–7 kcal mol^−1^ (ESI, Fig. S6†), where spontaneous re-protonation of Tyr413 occurred from protonated Lys241 with Tyr316 participating in the transfer (ESI, Fig. S1A and Movie S1†). We further analyzed the simulated trajectory and noted that the two energy maxima displayed in Fig. 2 correspond to the deprotonation of tyrosines, which are transiently stabilized by positively charged lysines (transient dipoles, see Movie S2†). Moreover, the proton transfer *via* tyrosines and serine occurs in a semi-concerted fashion, as also observed in other enzymes.^22,23^ These data not only allow us to identify the cause of energetic barrier in this region, but also that the proposed proton transfer route is a likely one.

Overall, our QM/MM MD simulations on high-resolution structure of *Y. lipolytica* complex I suggest that proton transfer from central Lys241 to terminal Lys383 occurs with reasonable energetics and involves protonation/deprotonation reactions of conserved tyrosine residues (Tyr316 and Tyr413), which are ideal candidates for site-directed mutagenesis in bacterial complexes.

### Horizontal proton transfer from conserved Lys/Glu pair to the conserved central lysine

The R region (Fig. 1B) has been suggested to catalyze proton transfer,^10^ but no charge transfer dynamics have been studied. Therefore, it remains unknown if proton migration on this path is energetically feasible or not. Explicit proton transfer in this section of the ND2 subunit is critical for those models of proton pumping which propose proton migration along the entire length of membrane arm of complex I.^6,10^ Therefore, we launched equilibrium unbiased QM/MM MD simulations of the R region with Lys241 modeled neutral (Tables S1 and S3†). Similar to the L region, we saw no spontaneous proton transfer from conserved Lys/Glu pair to the created hole position at Lys241, highlighting the presence of high energy barriers.

**Fig. 3:**
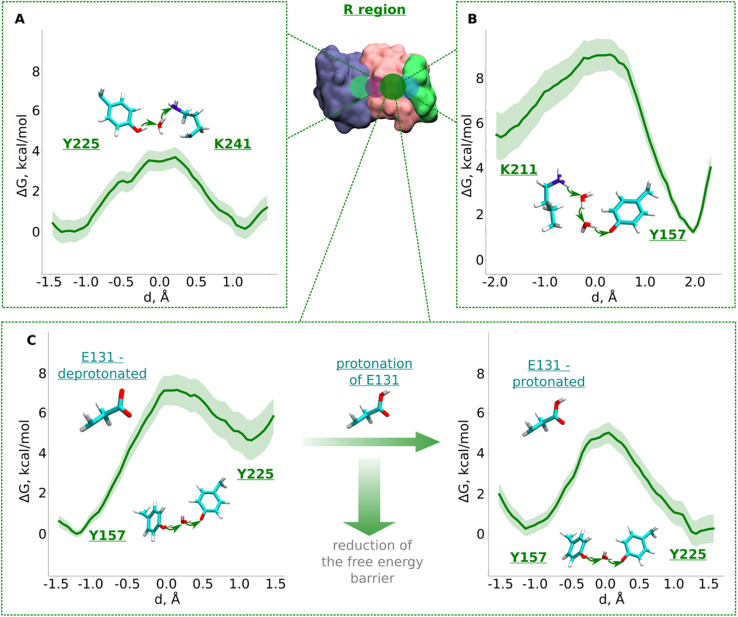
Free energy profiles of proton transfer from conserved Lys/Glu pair to central lysine (R region). (A) Tyr225 (neutral) → H_2_O → Lys241 (neutral) pathway. (B) Lys211 (protonated) → H_2_O → H_2_O →Tyr157 (deprotonated) pathway. (C) Proton transfer from Tyr157 (neutral) through H_2_O to Tyr225 (deprotonated). The left panel shows energetics when Glu131 of conserved Lys/Glu pair is deprotonated, and when protonated (right panel). The bold green lines are PMF profiles with light green shaded area highlighting the bootstrapping error range (see also methods). Occupational histograms for the reaction coordinates are given in ESI, Fig. S17B–E.† Inset shows ND4, ND2, and ND4L subunits in blue, pink, and green surface representation, respectively. The QM region where the proton transfer is studied is marked with the dark green circle.

Based on the critical role of tyrosine residues found above in the L region, we studied the proton transfer in R region in short-to-long fragments using QM/MM free energy simulations (Table S3† and setups 1–3,9). Proton transfer on the pathway connecting conserved Tyr225 to central Lys241 occurred with an activation energy barrier of ∼4.0 kcal mol^−1^ with equally favorable proton positions on both residues (Fig. 3A). However, despite protonation of Lys241 from Tyr225, subsequent propagation of the “hole” in the direction of Lys211 did not occur, which had now become “stuck” on Tyr225, suggesting the existence of activation energy barrier in the region from Tyr225 towards Lys/Glu pair (Fig. 1B and S1B, ESI†).

To investigate what will drive proton transfer from Lys211 of Lys/Glu pair, we initiated additional free energy simulations on the structurally-conserved Tyr157-H_2_O-Tyr225 pathway (Fig. 1B and S1B, ESI†), where Tyr225 was modelled anionic, and Tyr157 neutral (Table S3,† setup 2). The energy barrier we obtained was relatively higher (∼7.5 kcal mol^−1^) and, at the same time the protonation of Tyr225 (anionic) found to be highly unfavorable (Fig. 3C, left), in agreement with our above conjecture. We noted that in all these calculations, Glu131 of the conserved Lys/Glu pair was modelled deprotonated (ESI, Table S3†). However, surprisingly, both the kinetics and thermodynamics of proton transfer between the tyrosine residues substantially improved when Glu131 was modelled protonated (Fig. 3C, right). This is a strong indication that long range electrostatic effects are at play and proton transfer in this domain can be triggered by protonation of Glu131 of Lys/Glu pair, as long as the proton hole resides on Lys241 (Fig. 3C, see also ESI, Fig. S7†). p*K*_a_ calculations performed on simulation snapshots obtained from classical molecular dynamics indeed support a higher proton affinity of Glu131 (see Fig. S2† panel D). We additionally tested this notion by modelling an H_3_O^+^ ion (excess proton) between protonated Lys211 and neutral Tyr157. We found that when Glu131 was modelled protonated, a rapid proton transfer (in 50 fs) occurred to Lys241 in contrast to no such event when it was modelled anionic. Our simulation data directly show how protonation of conserved Lys/Glu ion pair can drive proton transfer in forward direction by reducing activation energy barriers.

The third pathway in the R region is located between Lys211 of the Lys/Glu pair and conserved Tyr157. There are several structurally-resolved water molecules in the pathway (Fig. 1B), and these are expected to support Grotthuss-like proton transfer. However, unbiased QM/MM MD simulations with Glu131 deprotonated showed no forward proton transfer from Lys211 to Tyr157. Instead, the proton was found to stabilize on Glu131, and was accompanied by the loss of the water path between Lys211 and Tyr157. Only when Glu131 was modelled neutral, we observed a water bridge (two H_2_O molecules) between Lys211 and Tyr157, yielding a rather low energy barrier (∼4 kcal mol^−1^) of protonation dynamics, with the proton position on Tyr157 much more favorable (Fig. 3B). We thus suggest that similar to the inter-tyrosine protonation dynamics (Fig. 3C, right panel), protonation of Tyr157 can be modulated by the charged state of Glu131 of Lys/Glu ion-pair that resides at a distance of ∼9 Å from the tyrosine.

**Fig. 4:**
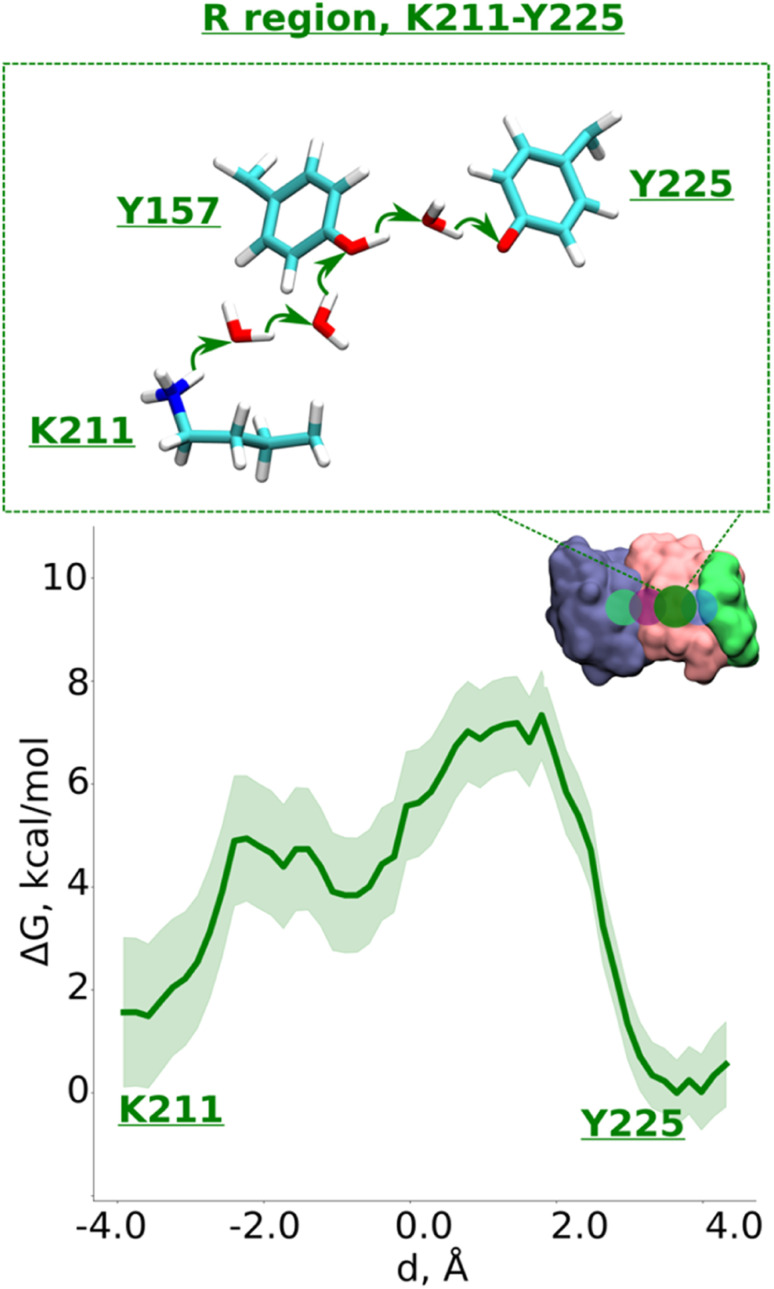
Proton transfer in the R region. Proton transfer occurs from Lys211 (protonated) → H_2_O → H_2_O→ Tyr157 (neutral) → H_2_O → Tyr225 (deprotonated) as depicted in the top panel. The bottom panel shows PMF (potential of mean force) profile of proton transfer derived from our QM/MM umbrella sampling simulations. Green shaded area around the bold green line depicts the bootstrapping error range (see also methods). Occupational histograms for the reaction coordinates are given in ESI, Fig. S17F.† The inset marks the studied QM region with dark green circle. The core membrane subunits ND4, ND2, and ND4L are shown in blue, pink, and green, respectively.

Data from classical MD simulations complement the picture that emerged from QM/MM MD simulations and show that protonated Lys211 sidechain drifts towards neutral Tyr157 upon protonation of Glu131/Glu66 pair residing at the interface (ESI, Fig. S2,† see more below). This behavior possibly facilitates the formation of a water path between Lys211 to Tyr157, which can benefit the proton transfer dynamics in this region. To track the energetics of proton transfer from Lys211 all the way to Tyr225, we performed an additional large-scale QM/MM free energy simulation with Glu131 protonated (see also Fig. S2†). It yielded an activation energy barrier of ∼6 kcal mol^−1^ with around 2 kcal mol^−1^ more favorable proton location on Tyr225 (Fig. 4; see also ESI, Figs. S8 and S9†). The metastable state at *d* ∼ −0.7 Å corresponds to the deprotonated Tyr157 prior its subsequent re-protonation and is in good agreement with the data from small-scale QM/MM free energy runs (Fig. 3B and C).

### Inter-subunit proton transmission in the membrane arm of complex I

**Fig. 5:**
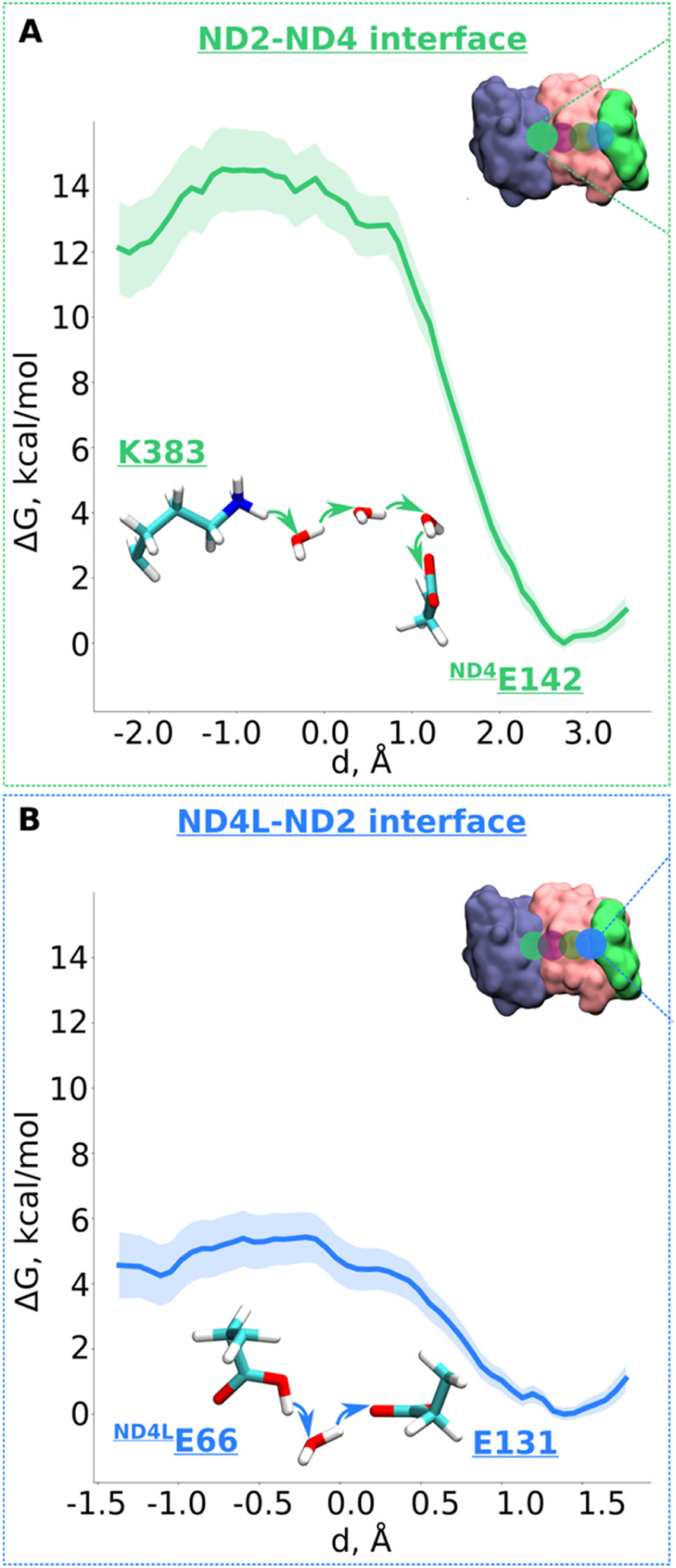
Free energy profiles of the proton transfer in the “interface regions”. (A) ND2-ND4 interface: Lys383 (protonated) → H_2_O → H_2_O → H_2_O → ^ND4^Gu142 (deprotonated) pathway. (B) ND4L-ND2 interface: ^ND4L^Glu66 (neutral) – H_2_O – Glu131 (deprotonated) pathway. The bold lines are PMF profiles with light shaded area highlighting the bootstrapping error range (see also methods). Occupational histograms for the reaction coordinates are given in ESI, Fig. S17G and H.† The inset depicts the core membrane subunits of respiratory complex I: ND4 (blue), ND2 (pink), and ND4L (green). The green (A) and blue (B) circles denotes the QM region where the proton transfer is investigated.

Next, we decided to estimate the protonation energetics at the subunit–subunit interface (ND2/ND4 and ND2/ND4L interfaces, see Fig. 1B). The high-resolution structure of mitochondrial complex I^10^ shows continuous water pathways between titratable residues residing at the subunit–subunit interface (Fig. 1B, S1C and D, ESI†). High-resolution structural data on complex I from other species also show similar interfacial hydration (ESI, Fig. S10†), suggesting that proton transfer can occur (see also ref. 24). Therefore, we next investigated the proton transfer dynamics on a chain of three water molecules spanning Lys383 and ^ND4^Glu142 *via* hydrogen bonds. PMF profiles obtained showed a low energy barrier of ∼3.5 kcal mol^−1^ with a much more favorable proton position on ^ND4^Glu142 (Fig. 5A and S11†). Also, for the ND4L-ND2 interface, we observed a very low barrier for proton transfer from ^ND4L^Glu66 to Glu131 of ∼1.5 kcal mol^−1^ with around 4 kcal mol^−1^ more advantageous proton location on Glu131 (Fig. 5B and S12, ESI†). Remarkably, this *driven* proton transfer caused ^ND4L^Glu30 (modelled neutral), which was outside the chosen US reaction coordinate (ESI, Fig. S1D and Movie S3†), to protonate and neutralize ^ND4L^Glu66, highlighting the cooperative nature of proton transfer dynamics, as observed also in other biological systems.^25^

To conclude, our multiscale simulation data reveal that inter- and intra-subunit proton transfer can occur with low activation energy barriers, and favorable thermodynamics. This has important implications on the proton pumping mechanism of complex I, as discussed below.

## Discussion

By applying QM/MM free energy simulations on a high-resolution structure of complex I, we have identified how a proton can migrate through the central hydrophilic axis of an antiporter-like subunit of complex I. According to our results, proton migration can occur with a hole-like mechanism with reasonable kinetics and thermodynamics. Four conserved titratable residues play a central role in catalyzing proton transfer in the antiporter-like subunits of complex I. However, our QM/MM simulations reveal that conserved tyrosine residues present in these membrane-bound subunits may play a key role in catalyzing proton transfer and are thus suggested here to be primary candidates for site directed mutagenesis studies to probe complex I proton pumping (see also ref. 10). The results also show a profound impact of the charge state of glutamic acid of conserved Lys/Glu pair. The protonation of Glu131 can reduce the energetic barriers of proton transfers between different residues, highlighting that long-range electrostatic effects are at play (see also ref. 6 and 16). Data from our QM/MM simulations also reveal that an explicit proton transfer can occur from conserved Lys/Glu pair to the central lysine, a notion that has not been investigated earlier. Furthermore, our calculations for the first time show that proton transfer can occur across interfaces between membrane-bound subunits with reasonable energetics. Based on these combined data, we discuss below a step-by-step mechanism of proton transfer in the ND2 subunit of complex I.

### Proton transfer mechanism in the antiporter-like subunit of complex I

According to the proton pumping mechanism proposed by Parey *et al.*,^10^ a pumped proton is released towards the central hydrophilic axis *via* the E channel upon electrostatic unlatching by quinone reduction and protonation at the second quinone binding site. This proton electrostatically drives the pumping of protons loaded on the antiporter-like subunits towards the P side *via* a single exit route present in terminal ND5 subunit, which has been studied biochemically, structurally, and computationally.^10,14,26^ Based on recent structural^6,10^ and computational data,^10^ the two other antiporter-like subunits ND2 and ND4 have been suggested to be devoid of P side proton exit routes (but see also, ^27^). However, they do uptake protons from the N side and E channel, thus catalyzing horizontal proton transfers parallel to the membrane.^10^

**Fig. 6:**
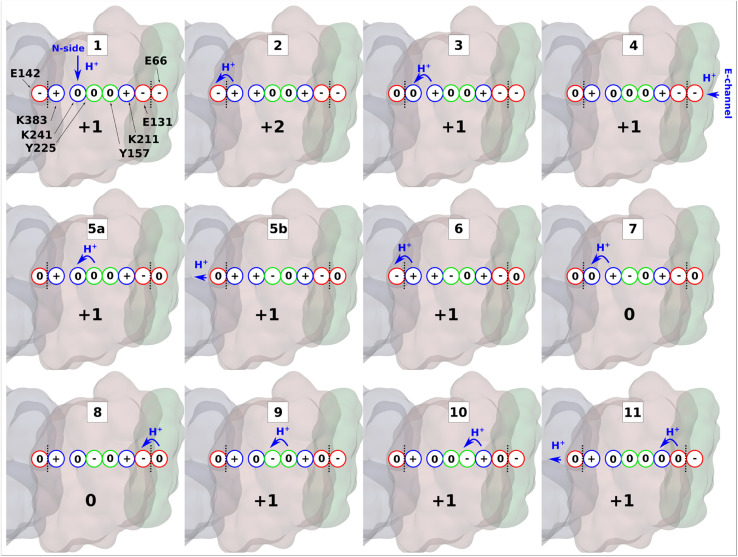
Proton transfer in the antiporter-like subunit of complex I. The proposed mechanism of lateral proton transfer in ND2 subunit of mitochondrial respiratory complex I, which is a direct continuation of the scheme proposed in ref. 10. Within one cycle shown here, the enzyme takes up one proton from the N-side (stage 1), and one from the E-channel (stage 4), and releases two protons to the ND4 subunit (stages 5b and 11). ND2 subunit is shown in brown, ND4 is in grey, and ND4L is in green. Red, blue, and green circles denote acidic, basic, and polar amino acids, respectively. A whole number at the bottom of each inset indicates the total charge of the depicted residues in ND2 subunit. Blue arrows show proton transfer and black dotted lines highlight separation between subunits.

To expand these conclusions, we propose the following mechanism of the lateral proton transfer in respiratory complex I (Fig. 6). Each antiporter-like subunit has a maximal proton/charge capacity, which is when achieved, requires an external trigger (such as an electrostatic push by protons released upon Q redox reaction^28^) causing the release of the proton to the neighboring subunit and so on so forth to the P side *via* ND5. Conversely, if the maximal proton/charge capacity is not reached, an antiporter-like subunit would accept protons from the bulk medium or neighboring subunits. As a starting point, we propose a proton transfer occurs from the N side of the membrane to the neutral Lys241 because of its high p*K*_a_ relative to the bulk (steps 1 to 2, changing charge of antiporter-like subunit from +1 to +2). The pathways observed in earlier MD simulations^14–16,24^ and recent high-resolution structural data support this scenario.^10^ Protonation of Lys241 causes excess charge to build up in ND2, which drives the release of a proton present on terminal Lys383 across ND2/4 interface to anionic ^ND4^Glu142 with a small barrier of ∼3.5 kcal mol^−1^ (Fig. 5A). The energetically favorable proton transfer across interface creates a proton vacancy on Lys383 (step 2).

In the next step, positively charged Lys241 donates its proton to terminal Lys383 (Fig. 2) causing a series of intra-subunit protonic rearrangements (step 3). Structural and classical MD simulation data suggested that the neutral state of central lysine is coupled with the closure of N-side connectivity by the movement of TMH11a/b and a conserved phenylalanine gate.^10^ At this stage, Lys241 waits for the second pumped proton to arrive *via* the horizontal axis from quinone binding tunnel. However, this requires proton transfers involving conserved tyrosine residues and does not occur until the second pumped proton arrives to ^ND4L^Glu66 (step 4). A cascade of forward proton transfers (steps 5–8) takes place causing total charge to change from +1 to 0 resulting in charge deficit in ND2 subunit. Inter-subunit proton transfer occurs from ^ND4L^Glu66 to Glu131 in step 8, which perturbs the inter-tyrosine protonation dynamics by long-range electrostatic effects (steps 8 to 9).

Finally, protonation of the anionic tyrosine (Tyr157) from Lys211 of Lys/Glu pair restores the system to ground state for a second cycle. In the cycle shown in Fig. 6, two pumped protons are taken up, one from the N-side (step 1) and one from the E-channel (step 4). In the proposed mechanism, protons diffuse through the entire horizontal axis of the antiporter-like subunit under the action of proton deficit (created by pumping) and excess (created by pumped proton released as part of Q redox chemistry). As part of this cycle, central lysine residues in antiporter-like subunits pick protons from the N side and may work cooperatively. Indeed, structural data revealed a state in which N side connectivity in ND2 was open, whereas the same path was closed in neighboring ND4 subunit.^10^ Overall, our results demonstrate that a lateral proton transfer can occur across the entire width of antiporter-like subunit of respiratory complex I *via* a Grotthuss-type “hole” scheme. This long range proton transfer in the membrane arm of complex I can be driven by protons injected from E channel as part of Q redox chemistry.^10,28^

## Methods

Hybrid quantum mechanical/molecular mechanical (QM/MM) simulations were performed on the high-resolution cryo-EM structure of mitochondrial complex I from *Yarrowia lipolytica* (PDB 7O71). The model system was limited to 6 core subunits: ND1, ND2, ND3, ND4, ND4L, ND6. The missing residues of the ND3 subunit were created by homology modelling using the MODELLER tool.^29^ All structural water molecules that were within 5 Å of the above-mentioned protein subunits were taken into account. To avoid the large-scale motions of the long terminus of ND3 subunit (residues 114 to 128), this region was excluded from the system. The QM/MM system encompassed almost 28 700 atoms.

To track the proton transfer energetics and dynamics, we performed biased and unbiased QM/MM MD simulations on the ND2 subunit that is shown to be most hydrated in the high-resolution structure of complex I.^10^ To enhance sampling and keep model systems computationally tractable, we split the horizontal axis of ND2 subunit (comprising both amino acids and structurally-resolved waters) into separate QM regions as part of QM/MM setup (see Table S1†). For biased QM/MM MD simulations, umbrella sampling (US) technique combined with weighted histogram analysis method (WHAM) were applied.^30^ QM/MM MD simulations were carried out using the combination of NAMD v2.14 (refs. ^31^ and 32) and ORCA 5.0.3 (ref. 33) software packages. After obtaining the structural coordinates from PDB, hydrogen atoms were added using PSFGEN VMD plugin.^34^ Amino acids were modelled in their standard protonation states unless otherwise mentioned (see below). Topotools VMD plugin was used for setting up the QM regions.^35^ The obtained model system was minimized at MM level for 200 steps with the steepest-descent algorithm, followed by QM/MM minimization for 500 steps.

In all simulations the temperature was maintained at 310 K using Langevin thermostat.^36^ Trajectories were obtained using Verlet integration method^37^ with a 1-fs timestep. No constraints were applied on covalent bonds to hydrogen atoms. Non bonded interactions were calculated using the Verlet^38^ scheme with a 12 Å cutoff, 10 Å switching, and 14 Å pairlist distance. QM part was treated with density functional theory (DFT) with hybrid B3LYP functional,^39^ and def2-SVP basis set.^40^ For additional testing and benchmarking purposes (see more below), CAM-B3LYP functional^41^ with def2-TZVP basis set^40^ was also used. To account for the long-range London dispersion interactions, the DFT-D3 dispersion correction^42^ was applied. The SCF energy tolerance was set to 10 × 10^−8^ au. Coulomb interaction between QM and MM parts of the system was implemented using additive electrostatic embedding scheme.^32^ To describe the MM framework, CHARMM36 (ref. 43) force field was used.

To perform US free energy simulations, we used the linear combination of the pathway-forming atomic bonds as a reaction coordinate *d* (ESI, Fig. S13†):

1 *d* = *d*_1_ − *d*_2_ + *d*_3_ − *d*_4_

In the first simulation window, the reaction coordinate was restrained to its initial position (*d*_0_) by a harmonic potential:2
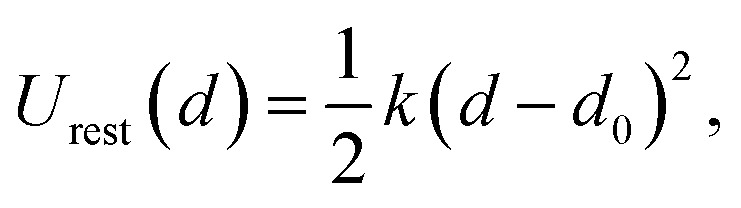
where the force constant *k* was chosen to be equal to 100 kcal mol^−1^ Å^−2^. Every next window was created by shifting *d*_0_ by Δ*d* = 0.2 Å with the velocity 10 Å ps^−1^. This procedure was implemented with the “colvars” NAMD module.^32,44^ After the generation procedure, each window was equilibrated for 1.8 ps, followed by a 2-ps production run further utilized for free energy analysis. The PMF profiles were calculated with WHAM analysis tool.^45^ The bootstrapping errors were obtained using 1000 Monte-Carlo trials with a 50-fs correlation time.

The total QM/MM MD simulation time in this work (including unbiased runs, and all US windows) is ∼1.5 ns. The VMD software package was used for the trajectory analysis and visualization.^34^

To investigate the effect of density functional and basis set on free energy profiles, we performed free energy calculations on the pathways Tyr157-H_2_O-Tyr225 (R region), and ^ND4L^Glu66-H_2_O-Glu131 (ND4L–ND2 interface) using CAM-B3LYP density functional and def2-TZVP basis set (ESI, Table S1 and Fig. S14, S15†). Only a minor difference in activation energy barrier (∼1.5 kcal mol^−1^ higher) was observed, which suggests the choice of B3LYP density functional and def2-SVP basis set is reasonable for studying proton transfer dynamics and energetics. Moreover, this level of theory was previously validated in extensive QM/MM studies of proton transfer in similar biological systems.^46–48^

Classical atomistic MD simulations were also performed on the complete structure of complex I from *Yarrowia lipolytica* (PDB:7O71). A detailed description of the model system setup can be found in ref. 10. Briefly put, the simulations were performed in two charged states of the protein; PN1 – in which titratable amino acids (Lys, Glu, Asp and Arg) were modeled in their charged states, whereas in state PN2 – protonation states of these amino acids were defined based on p*K*_a_ calculations, which were performed with the PROPKA software.^19,49^ In the current work, all three simulation replicas of PN1 and PN2 states were extended to 1 μs using the same simulations conditions and GROMACS v2020.3 package.^50^ In another state (PN3), a ∼150 ns MD simulation was performed with ND2 residues Lys383 and Glu131 modelled neutral. Analysis of the simulation trajectories was performed using VMD software.^34^ RMSF (Fig. S16†) of key residues constituting the proton transfer path remains low (especially in PN2 state) and residues do not show large scale conformational changes from the observed structural conformation in microseconds time scales (1 μs × 3 replicas). This also supports the usage of high-resolution structural data for QM/MM MD simulations performed here.

## Data availability

All the simulation trajectories and analysis data are available from the corresponding author upon reasonable request.

## Author contributions

OZ performed QM/MM simulations, analyzed data, prepared figures, and wrote the initial draft of the manuscript. AD analyzed data, prepared figures, movies, and contributed to the discussions. JL performed classical MD simulations and contributed to manuscript writing. VS designed and supervised the research, prepared figures, wrote and refined the manuscript.

## Conflicts of interest

The authors declare no competing interests.

## Supplementary Material

SC-014-D3SC01427D-s001

SC-014-D3SC01427D-s002

SC-014-D3SC01427D-s003

SC-014-D3SC01427D-s004
