# Supplementary material for: Horizontal proton transfer across the antiporter-like subunits in mitochondrial respiratory complex I
Source: Chem Sci. 2023 May 10;14(23):6309–18. doi: 10.1039/d3sc01427d (PMC10266447; doi:10.1039/d3sc01427d)
Supplement: SC-014-D3SC01427D-s003 [file SC-014-D3SC01427D-s003.pdf]

Electronic Supplementary Material (ESI) for Chemical Science.
This journal is © The Royal Society of Chemistry 2023

Supplementary Information

Horizontal proton transfer across the antiporter-like subunits in mitochondrial respiratory complex I

Oleksii Zdorevskyi[1], Amina Djurabekova[1], Jonathan Lasham[1], Vivek Sharma[1,2,*]

[1]Department of Physics, University of Helsinki, Helsinki, Finland

[2]HiLIFE Institute of Biotechnology, University of Helsinki, Helsinki, Finland

*To whom correspondence should be addressed: vivek.sharma@helsinki.fi

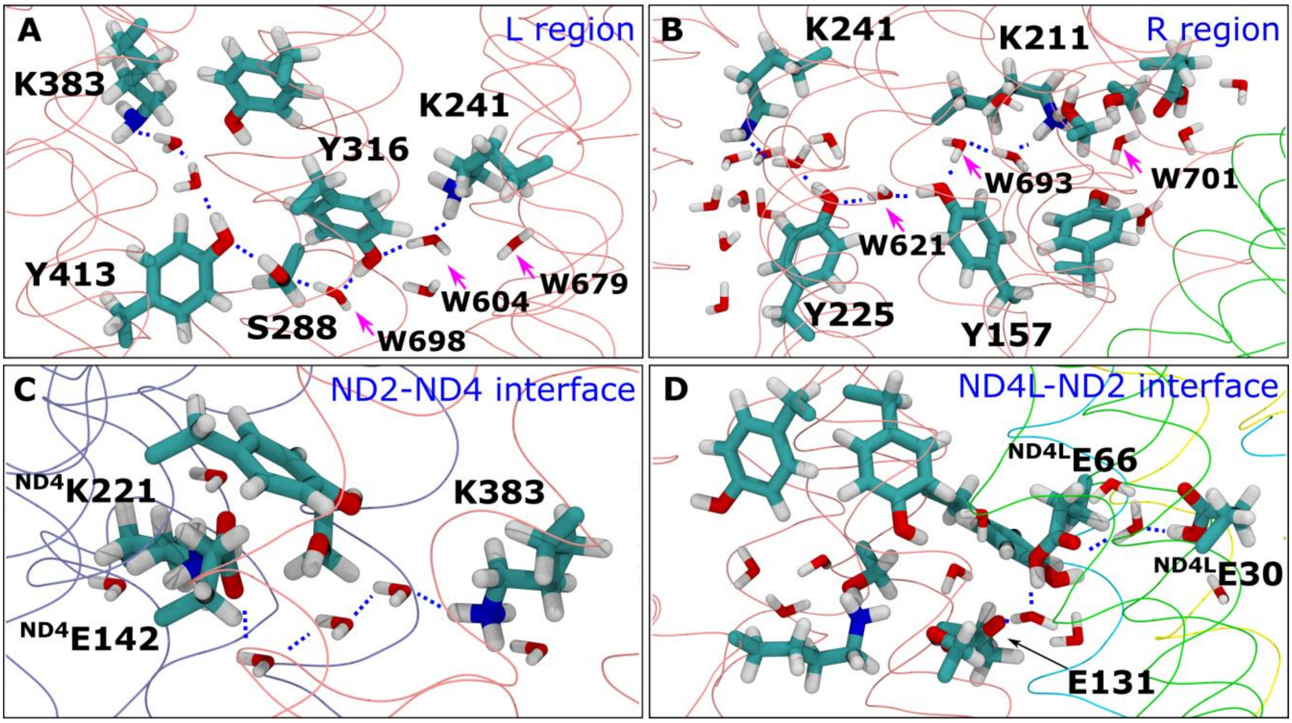

*Figure S1. QM regions studied in this work: (**A**) L region. (**B**) R region. (**C**) ND2-ND4 interface. (**D**) ND4L-ND2 interface. Pink arrows indicate the water molecules replaced by H_3O^+ ion in selected setups (Tables S2, S3). Amino acid and water residue ids are based on PDB entry: 7O71.*

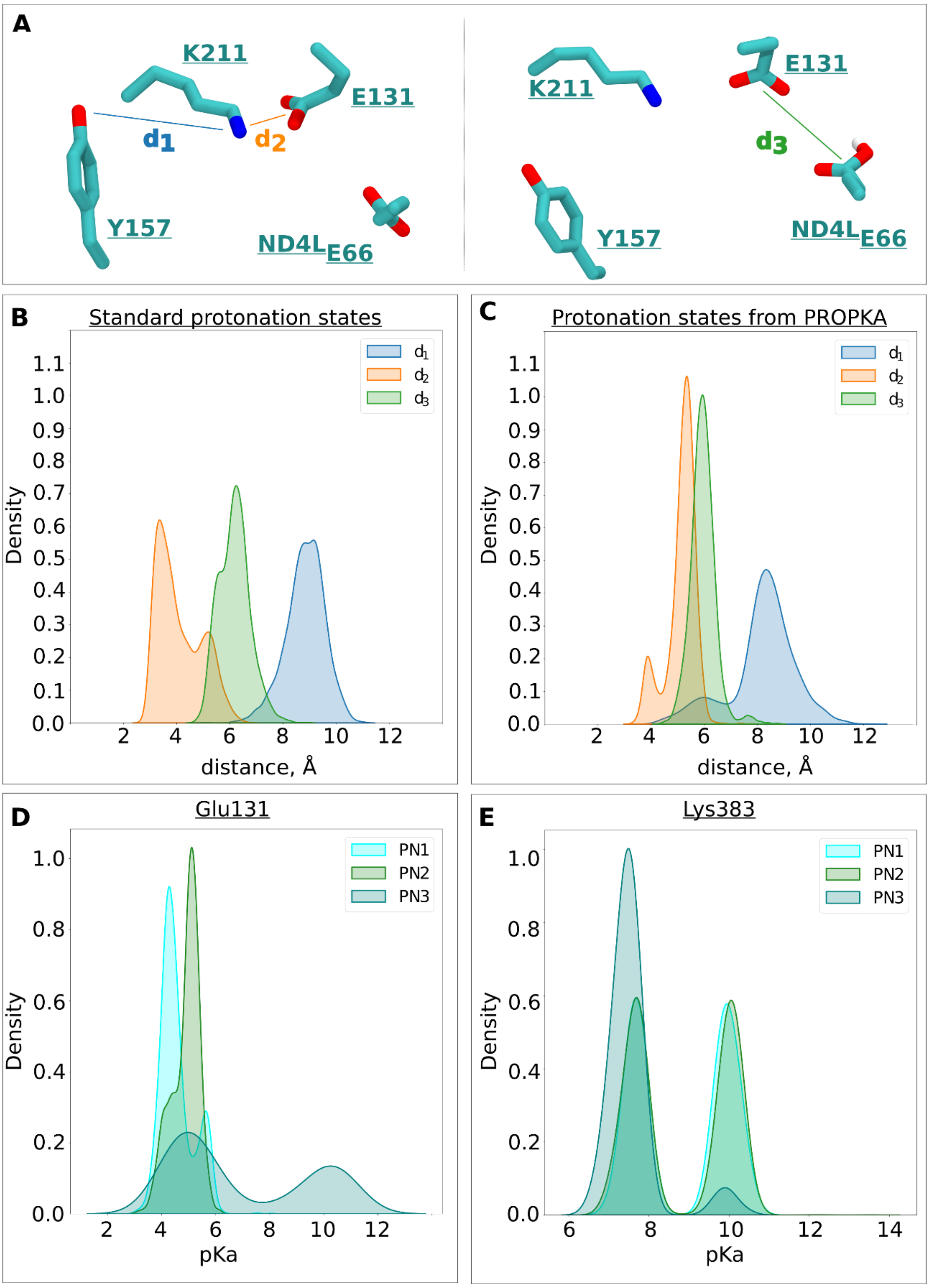

*Figure S2. Sidechain dynamics in different protonation states of ND2 residues. (**A**) Distances between titratable residues analyzed in classical MD simulations: d_1 (in cyan) - distance from of Lys211 (sidechain nitrogen) to Tyr157 (sidechain oxygen); d_2 (in orange) - from Lys211 (sidechain nitrogen) to Glu131 (sidechain Cε); d_3 (in green) - distance between the Cε atoms of Glu131 and ^{ND4L}Glu66. (**B, C**) Distributions of the distances d_1, d_2, and d_3 in the trajectories with standard protonation states (**B**), and protonation states obtained from PROPKA calculations (^{ND4L}Glu66 protonated, **C**). (**D, E**) Distributions of the pK_a values of Glu131 (**D**) and Lys383 (**E**) of ND2 subunit obtained from simulations performed in different states (see methods). Panels (**B-E**) display smoothened histograms by means of kernel density estimation method (available in Python).*

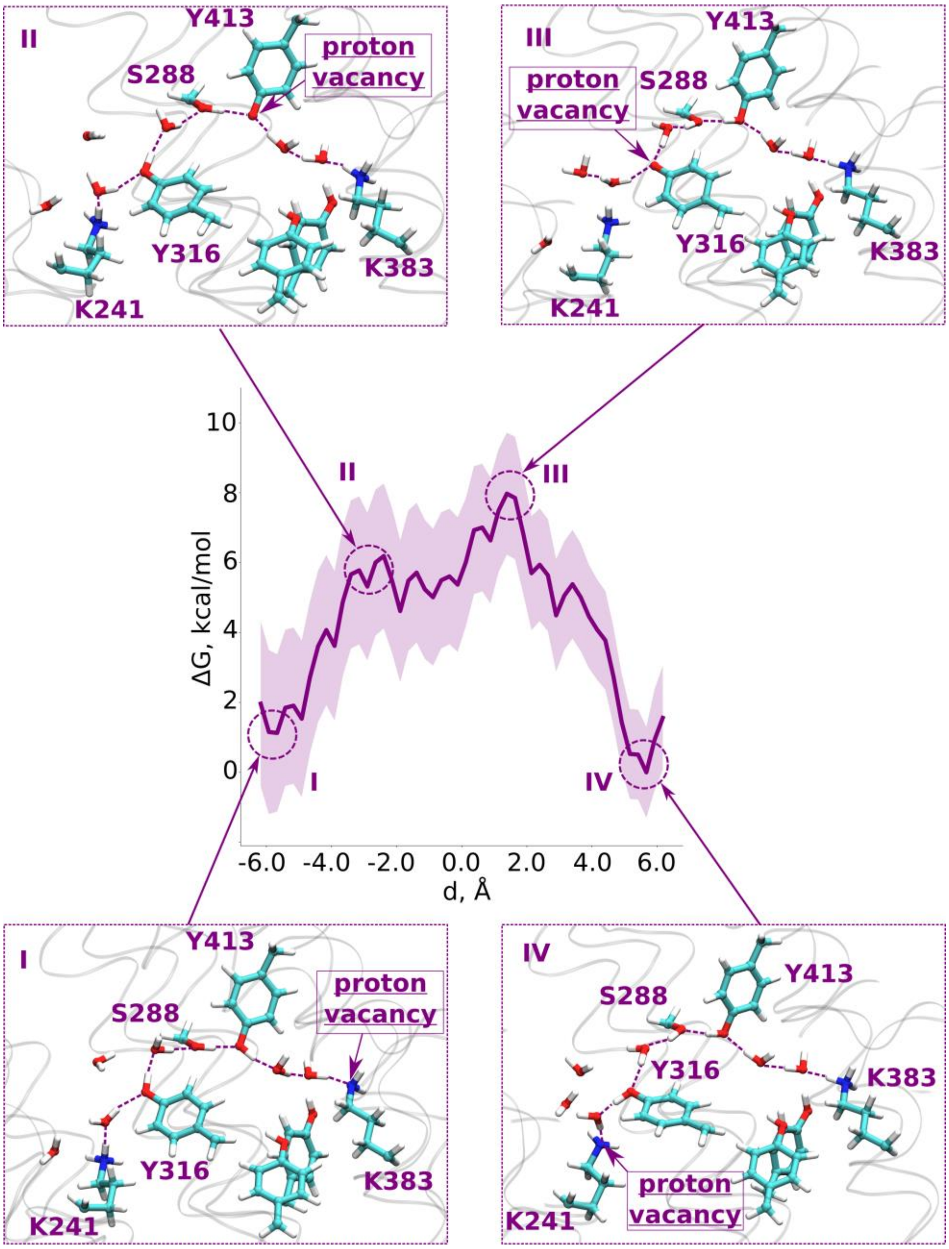

Figure S3. Free energy profile of the proton transfer from central to terminal lysine in the L region of the ND2 subunit: Lys241 (protonated)→H_2O→Tyr316 (neutral)→H_2O→Ser288 (neutral)→Tyr413 (neutral)→H_2O→H_2O→Lys383 (neutral). Insets show the snapshots from umbrella sampling simulation windows corresponding to the respective parts of the free energy profile. Dashed lines illustrate the hydrogen-bond network along the proton transfer pathway.

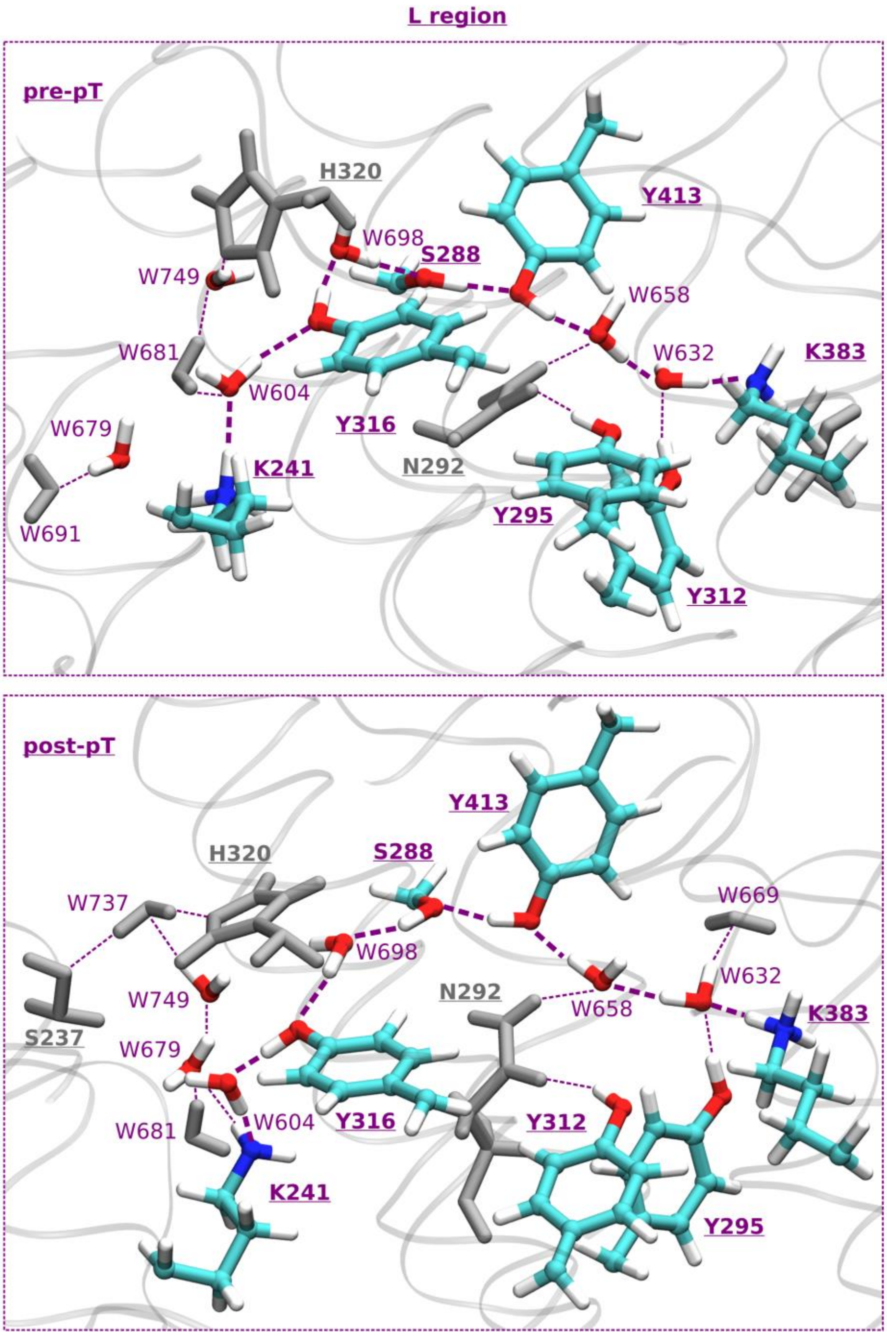

Figure S4. Hydrogen bond patterns for the proton transfer pathway in the L region of the ND2 subunit: Lys241 (protonated)→H_2O→Tyr316 (neutral)→H_2O→Ser288 (neutral)→Tyr413 (neutral)→H_2O→H_2O→Lys383 (neutral). The top panel shows the initial state before proton transfer (corresponding to state I in Fig. S3), whereas the bottom panel illustrates the post-pT state (state IV in Fig. S3). Protein sidechains and H_2O molecules from the MM region forming hydrogen bonds with QM residues are shown in grey. For visualization of hydrogen bonds, the distance cutoff between the heavy atoms (O...O, N...N) is chosen 3.5 Å, and for the angle O-H...O (N-H...O, O-H...N) the cutoff is 30 degrees. Hydrogen bonds forming the proton transfer pathway are marked with thick dashed lines. For a similar pre and post proton transfer hydrogen bond analysis, see (1).

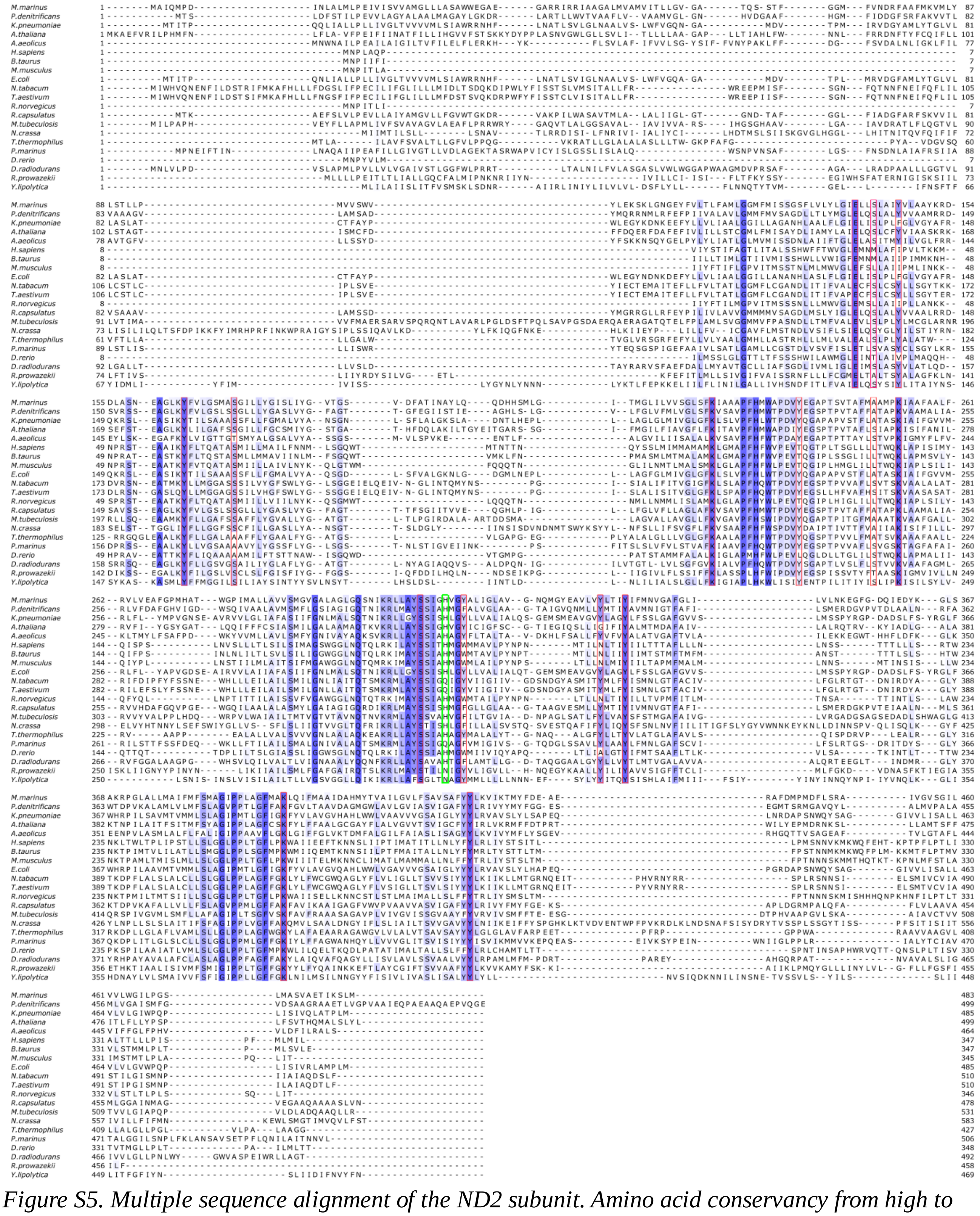

Figure S5. Multiple sequence alignment of the ND2 subunit. Amino acid conservancy from high to low is shown in the shade of blue (dark to light). The red boxes represent residues that are a part of the QM region. The green box highlights the Asn292 residue in ND2 subunit of Y. lipolytica that corresponds to His186 in M. musculus. The sequence alignment was generated using Mafft (2) and visualized with Jalview (3).

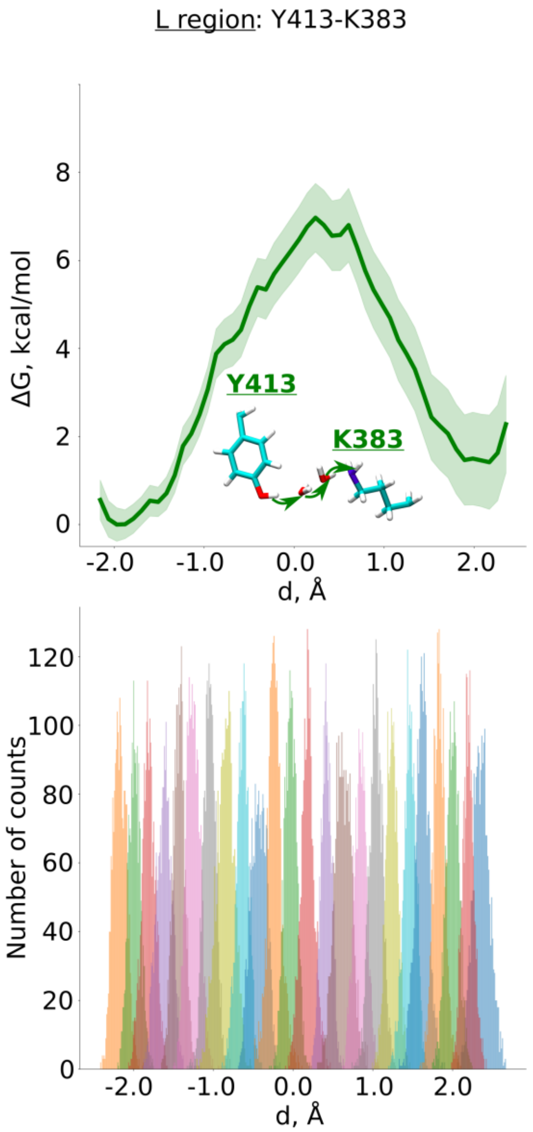

Figure S6. Free energy profile (top) and occupational histograms (bottom) for proton transfer from Tyr413 to Lys383 in the L region.

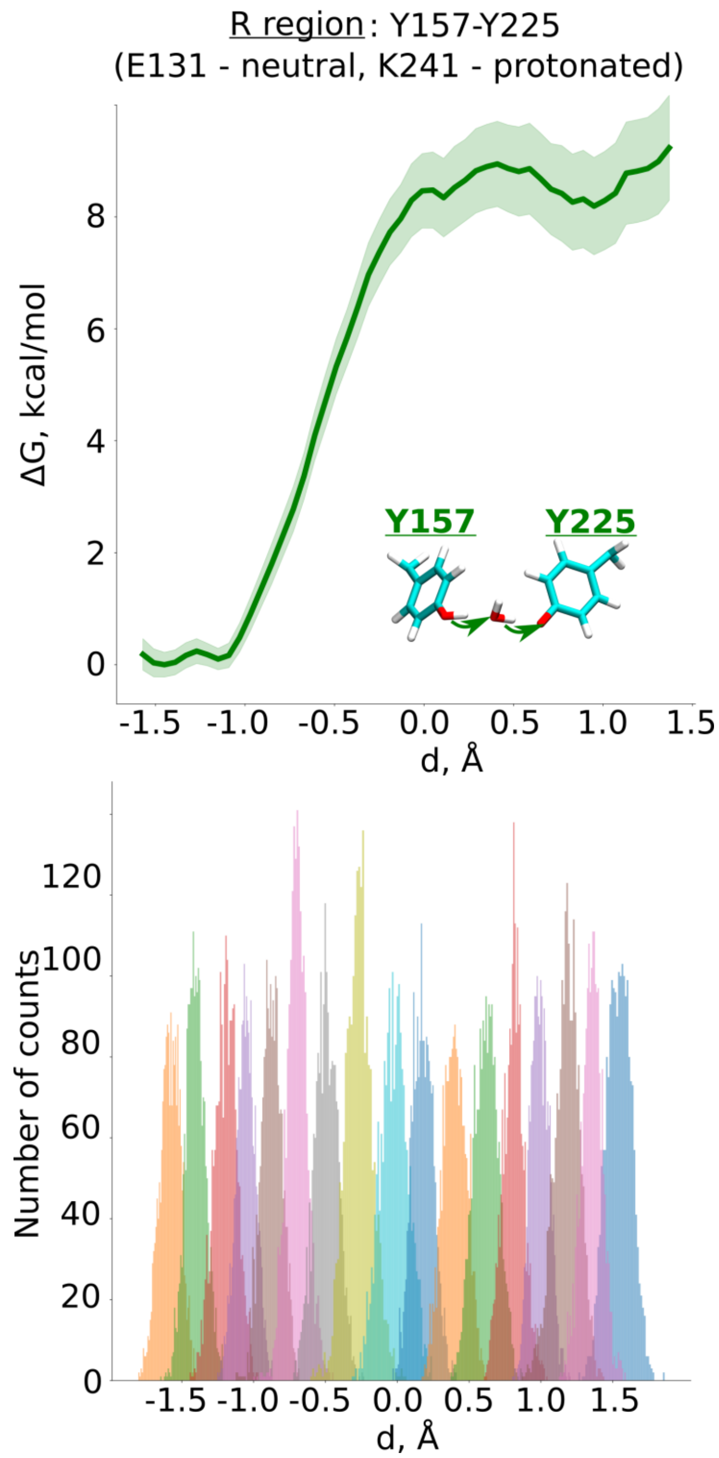

Figure S7. Free energy profile (top) and occupational histograms (bottom) for proton transfer from Tyr157 (neutral) to Tyr225 (deprotonated), when Glu131 is protonated, and Lys241 is also protonated.

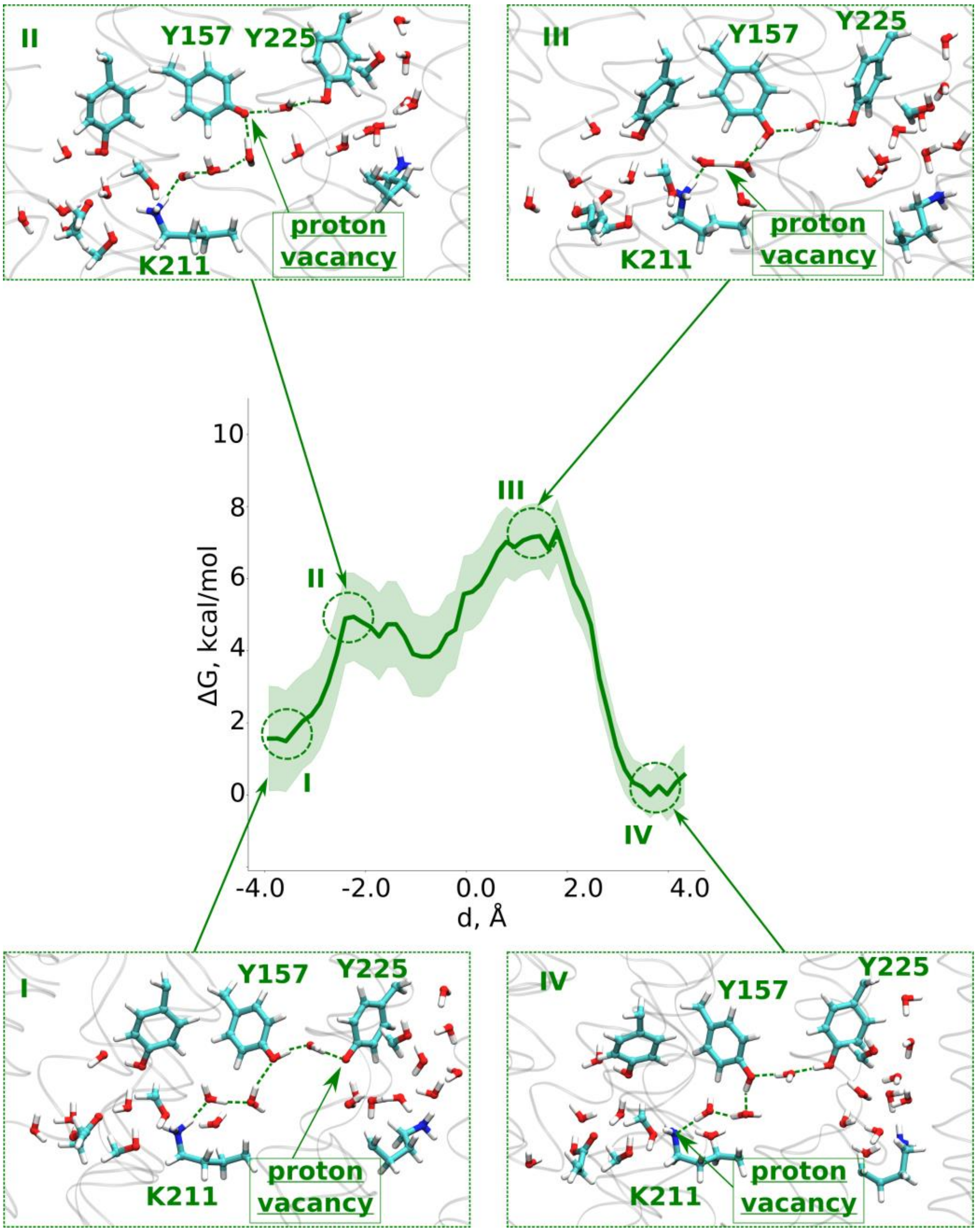

Figure S8. Free energy profile of the proton transfer in the R region of the ND2 subunit: Lys211 (protonated)→H_2O→H_2O→Tyr157 (neutral)→H2O→Tyr225 (deprotonated). Insets show the snapshots from umbrella sampling simulation windows corresponding to the respective parts of the free energy profile. Dashed lines illustrate the hydrogen-bond network along the proton transfer pathway. Licorice representation depicts the protein residues and water molecules included in the QM region.

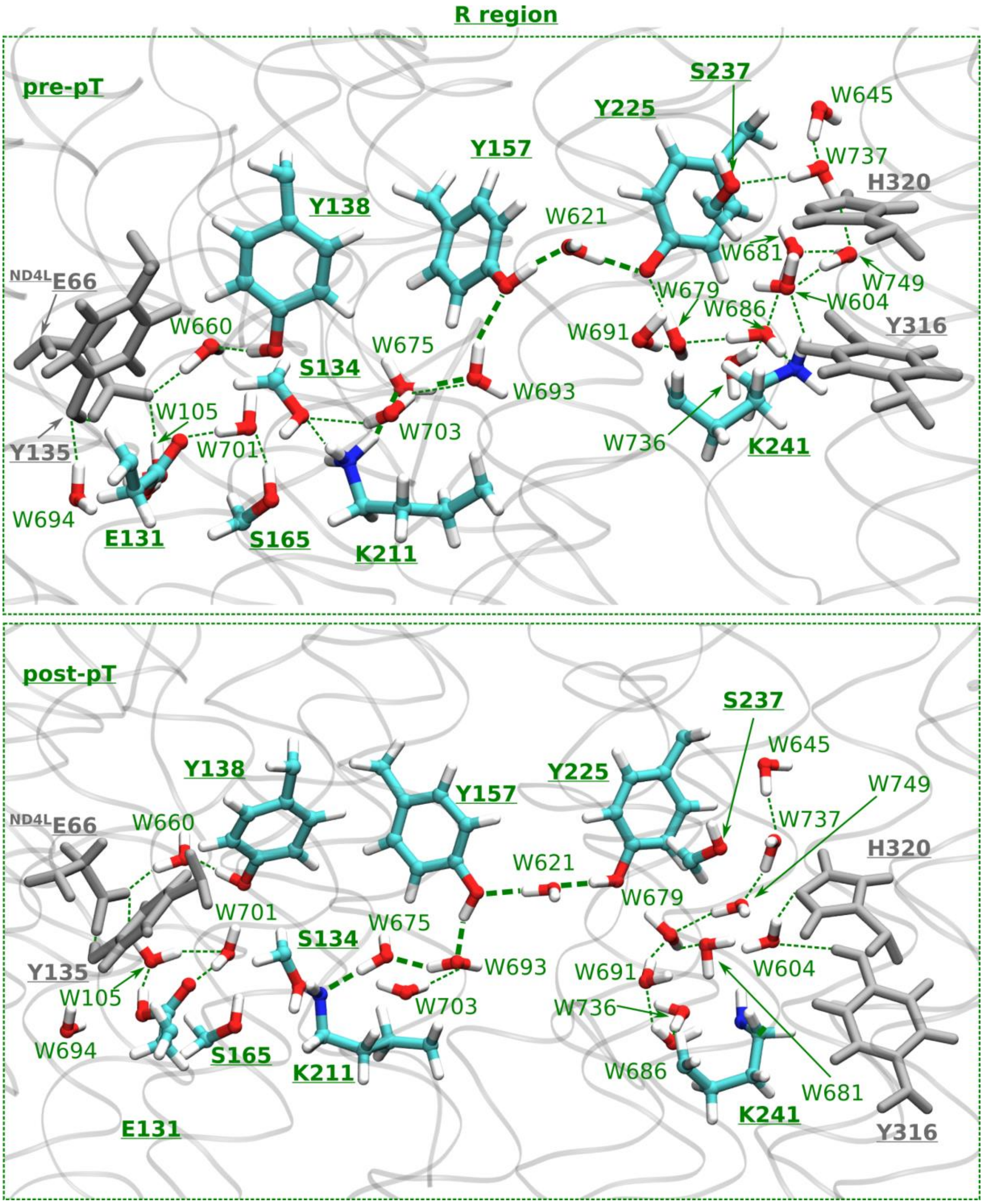

Figure S9. Hydrogen bond patterns for the proton transfer pathway in the R region of the ND2 subunit: Lys211 (protonated)→H_2O→H_2O→Tyr157 (neutral)→H_2O→Tyr225 (deprotonated). The top panel shows the initial state before the proton transfer (corresponding to state I in Fig. S8), whereas the bottom panel illustrates the post-pT state (state IV in Fig. S8). For visualization of hydrogen bonds, the distance cutoff between the heavy atoms (O...O, N...N) is chosen 3.5 Å, and for the angle O-H...O (N-H...O, O-H...N), the cutoff is 30 degrees. Protein sidechains and H_2O molecules from the MM region forming hydrogen bonds with the QM residues are shown in grey. Hydrogen bonds forming the proton transfer pathway are marked with thick dashed lines. For a similar pre and post proton transfer hydrogen bond analysis, see (1).

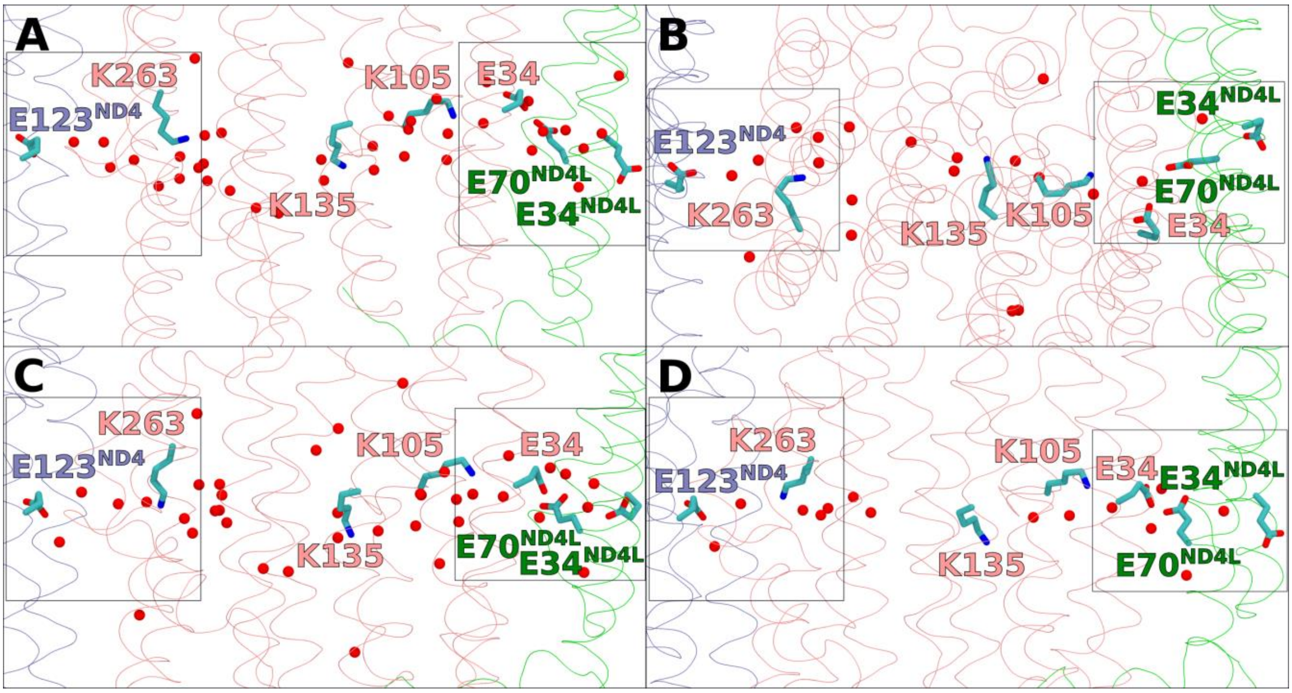

Figure S10. Interfacial hydration between subunits ND4L, ND2 and ND4. Data is shown from cryo-EM structures of mitochondrial respiratory complex I from different organisms: ***A*** *- Sus scrofa (PDB entry: 7vc0);* ***B*** *- Escherichia coli (PDB entry: 7z7v);* ***C*** *- Ovis aries (PDB entry: 6zka);* ***D*** *- Ovis aries (PDB entry: 6zkb).*

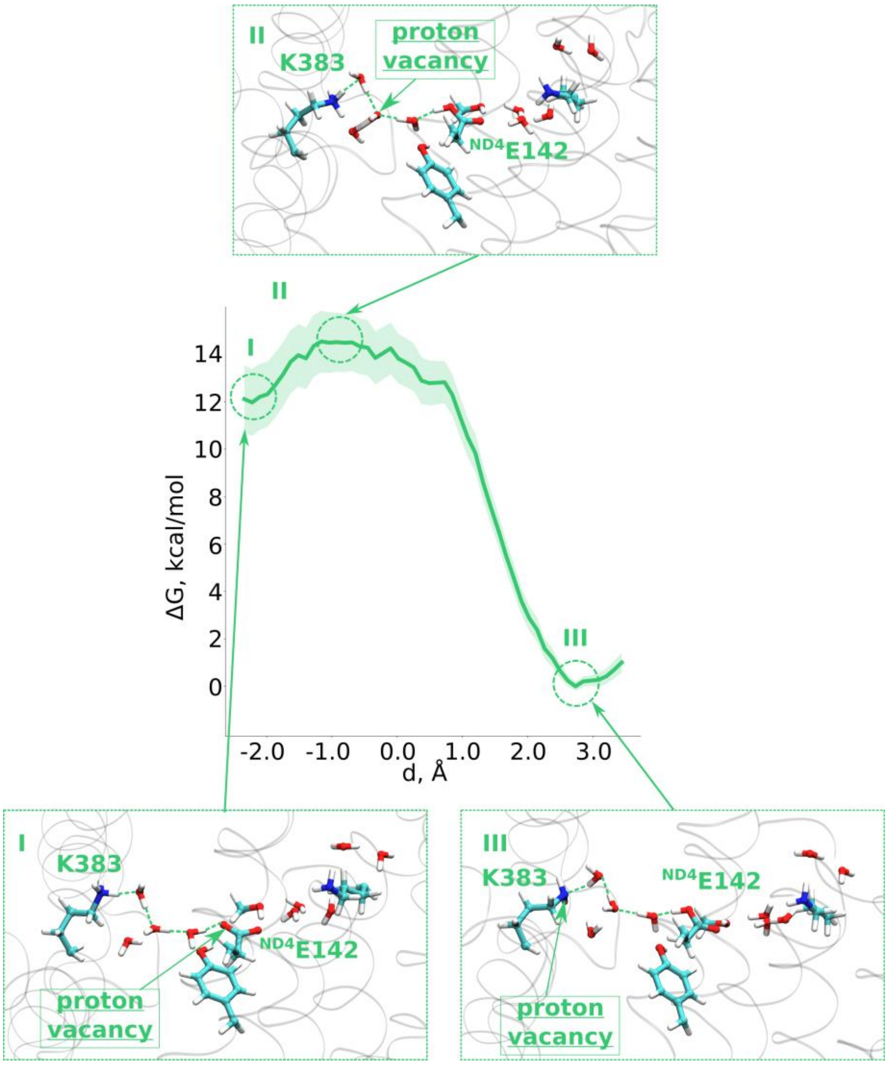

Figure S11. Free energy profile of the proton transfer in the interface region between the ND2 and ND4L subunits: ^{ND4L}*Lys383 (protonated)→*H_2O*→*H_2O*→*H_2O*→Glu142 (deprotonated). Each part of the free energy profile is additionally illustrated by the snapshots from umbrella sampling simulation windows. Dashed lines denote the hydrogen-bond network along the proton transfer pathway. Protein residues and water molecules included in the QM region are shown in licorice representation.*

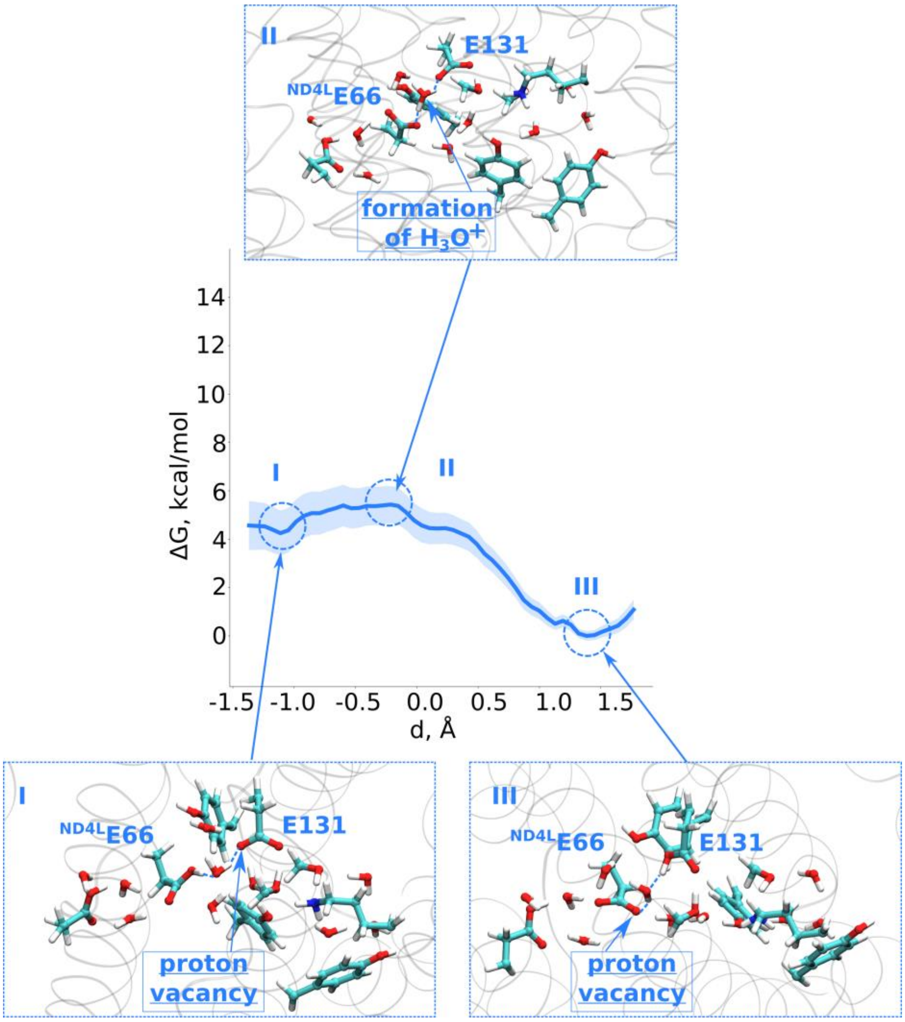

Figure S12. Free energy profile of the proton transfer in the interface region between the ND4L and ND2 subunits: ^{ND4L}Glu66 (protonated)→H_2O→Glu131 (deprotonated). Each part of the free energy profile is additionally illustrated by the snapshots from umbrella sampling simulation windows. The hydrogen-bond network along the proton transfer pathway is marked by the dashed lines. Protein residues and water molecules included in the QM region are shown in licorice.

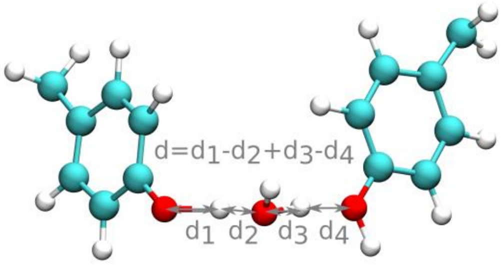

Figure S13. Definition of the reaction coordinate in QM/MM umbrella sampling MD simulations.

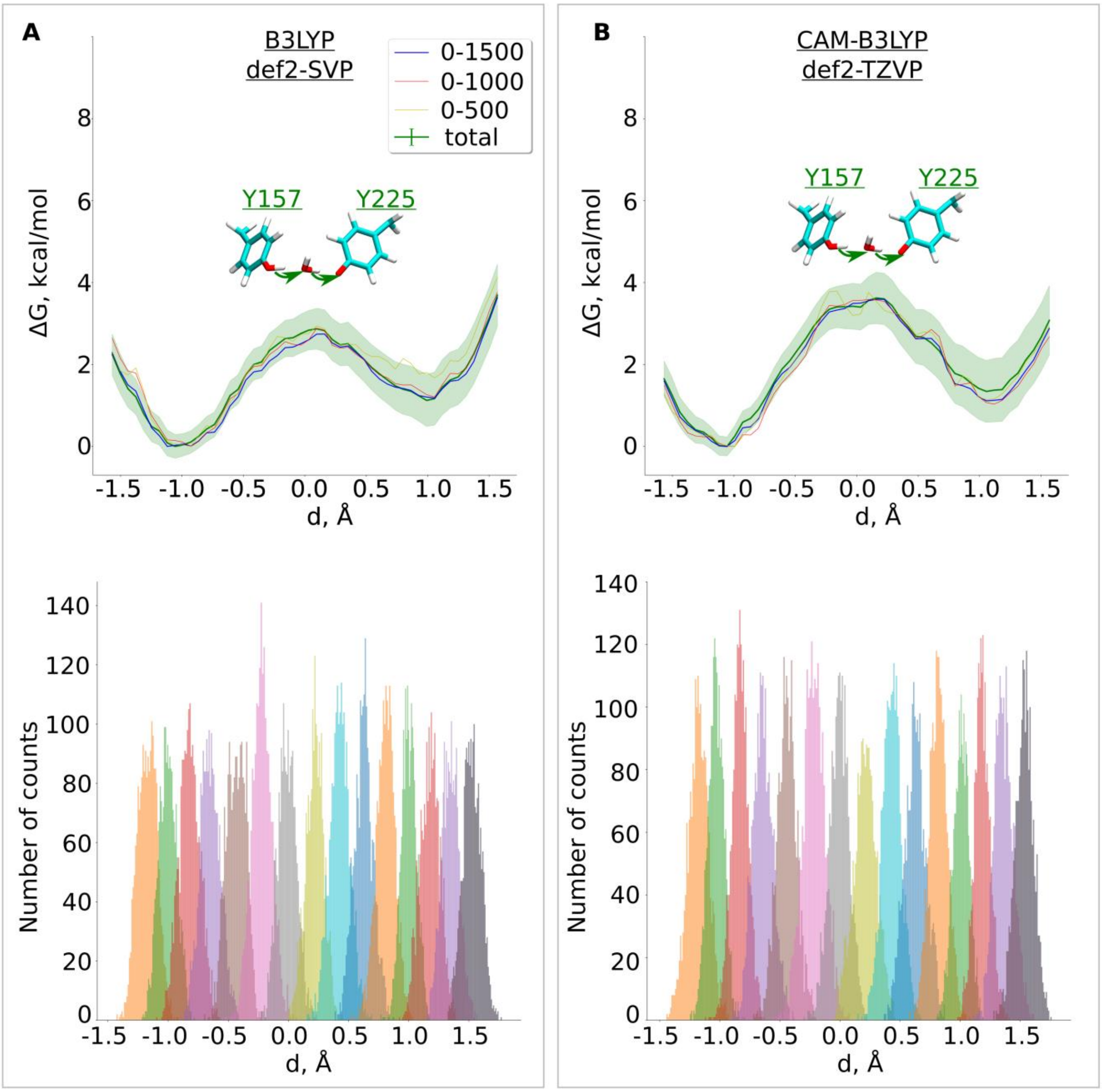

*Figure S14. Free energy profiles (top) and occupational histograms (bottom) for the proton transfer pathway from Tyr157 (neutral) to Tyr225 (deprotonated). (**A**) B3LYP functional with def2-SVP basis set; (**B**) CAM-B3LYP functional with def2-TZVP basis set. In these setups, QM region consists exclusively of the atoms shown in the insets of the top panels (Table S1). Colored lines correspond to the partial contributions to free energy profile from the first 500 fs (brown), 1000 fs (red), and 1500 fs (blue). The error bars (shaded green) are based on bootstrapping errors calculated from the whole 2-ps trajectory.*

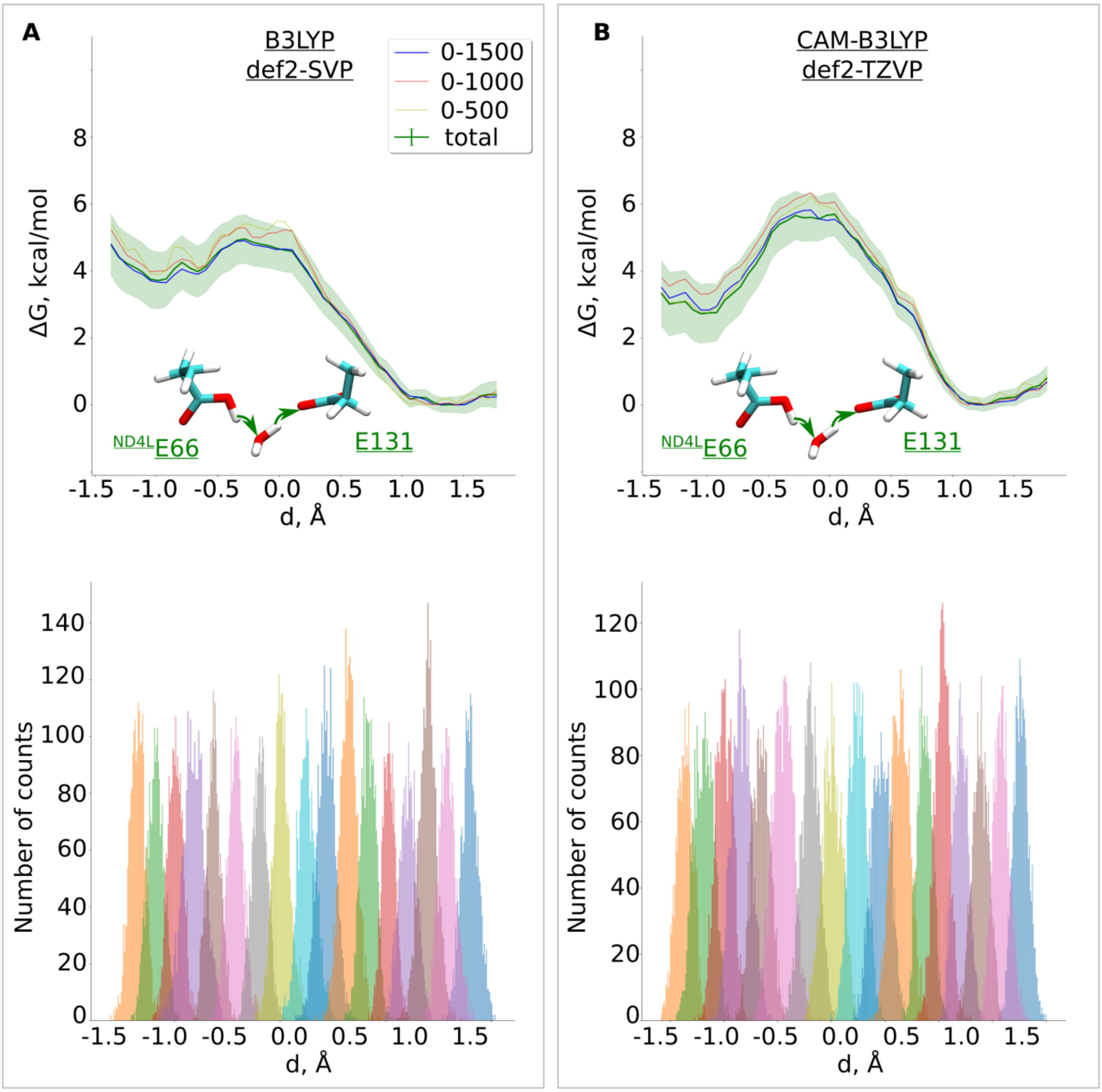

*Figure S15. Free energy profiles (top) and occupational histograms (bottom) for the proton transfer pathway from ^{ND4L}Glu66 (protonated) to Glu131 (deprotonated): (**A**) B3LYP functional with def2-SVP basis set; (**B**) CAM-B3LYP functional with def2-TZVP basis set. In these setups, QM region consists exclusively of the atoms shown in the insets of the top panels (Table S1). Colored lines correspond to the partial contributions to the free energy profile from the first 500 fs (brown), 1000 fs (red), and 1500 fs (blue). The error bars (shaded green) are based on bootstrapping errors calculated from the whole 2-ps trajectory.*

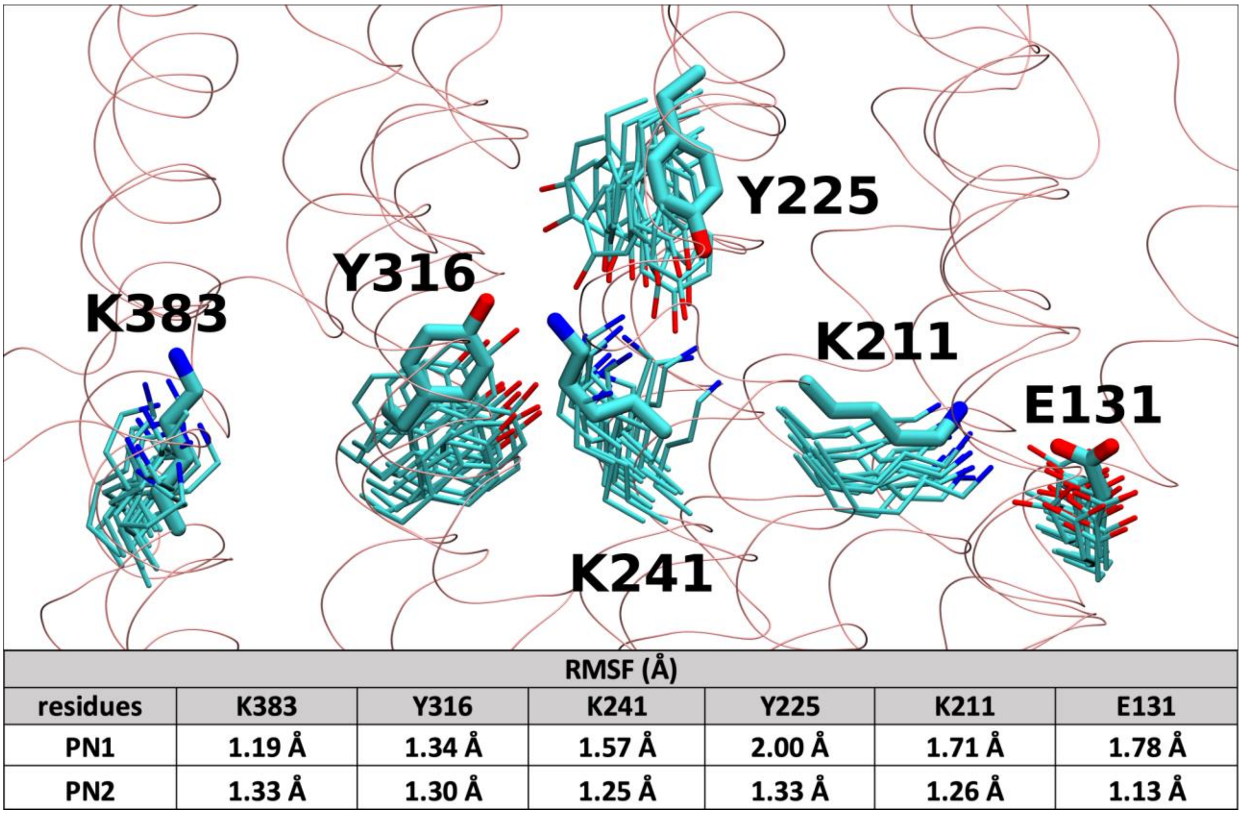

RMSF (Å)						
**residues**	**K383**	**Y316**	**K241**	**Y225**	**K211**	**E131**
**PN1**	**1.19 Å**	**1.34 Å**	**1.57 Å**	**2.00 Å**	**1.71 Å**	**1.78 Å**
**PN2**	**1.33 Å**	**1.30 Å**	**1.25 Å**	**1.33 Å**	**1.26 Å**	**1.13 Å**

Figure S16. Fluctuations of the titratable residues of the central hydrophilic axis of the ND2 subunit, derived from classical MD simulations from setups PN1 and PN2. The figure was obtained by combining the trajectory snapshots from all of the three replicas of PN2 setup with a stride of 200 frames. The table in the bottom panel shows the RMSF (root mean square fluctuation in Å) values of each of the considered residues in the mentioned setups.

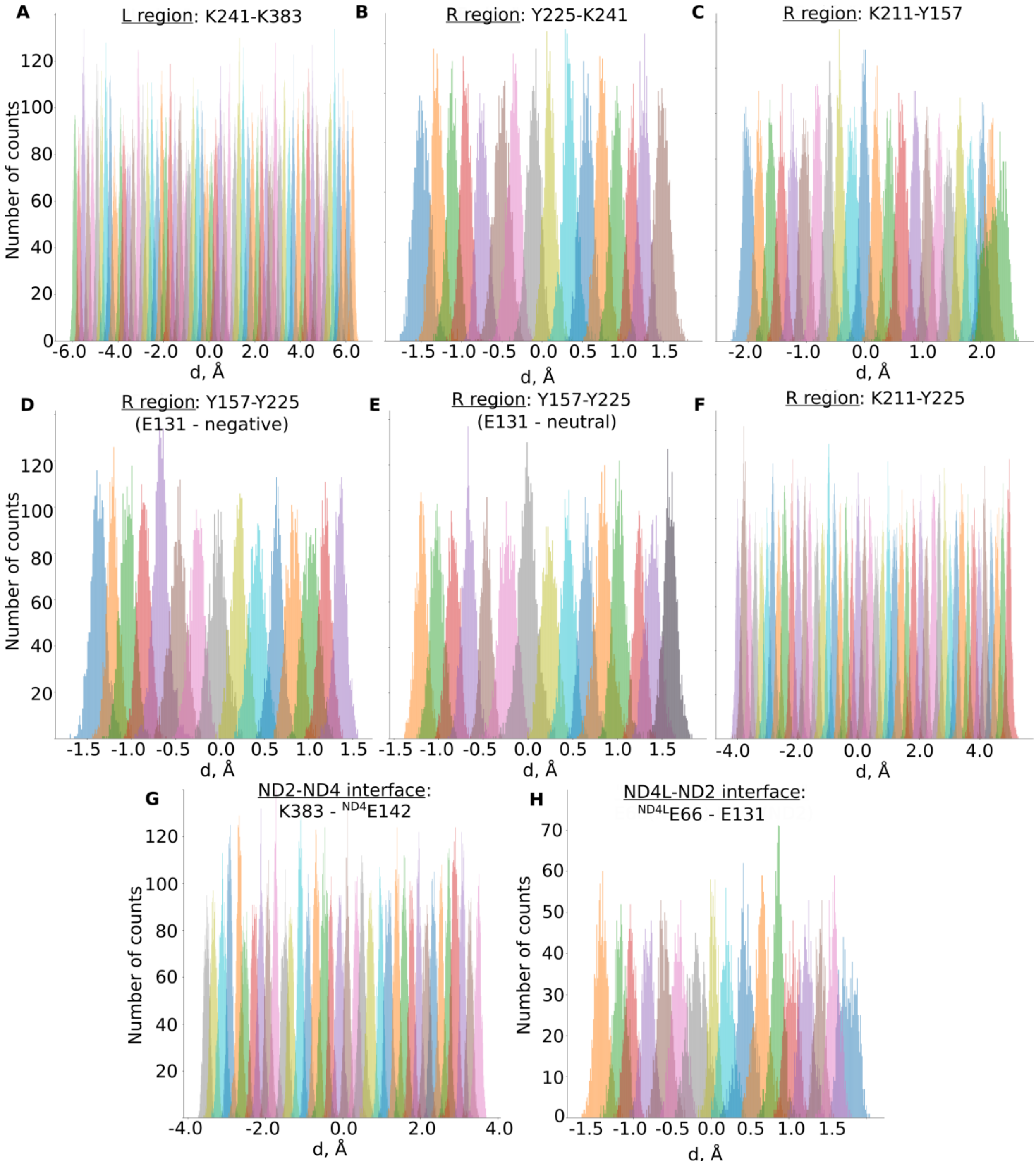

Figure S17. Occupational histograms from QM/MM US simulations: Proton transfer pathway ***(A)*** *Lys241 (protonated)→H_2O→Tyr316 (neutral)→H_2O→Ser288 (neutral)→Tyr413 (neutral)→H_2O→H_2O→Lys383 (neutral), L region (Fig. 2).* ***(B)*** *Tyr225 (neutral)→H_2O→Lys241 (neutral), R region (Fig. 3A).* ***(C)*** *Lys211 (protonated)→H_2O→H_2O→Tyr157 (deprotonated), R region (Fig. 3B).* ***(D)*** *Tyr157 (neutral) through H_2O to Tyr225 (deprotonated), when Glu131 is deprotonated, R region (Fig. 3C, left panel);* ***(E)*** *Tyr157 (neutral) through H_2O to Tyr225 (deprotonated), when Glu131 is protonated, R region (Fig. 3C, right panel).* ***(F)*** *Lys211 (protonated)→H_2O→H_2O→Tyr157 (neutral)→H_2O→Tyr225 (deprotonated), R region (Fig. 4).* ***(G)*** *Lys383 (protonated) → H_2O → H_2O → H_2O → ^{ND4}Gu142 (deprotonated), ND2-ND4 interface (Fig. 5A).* ***(H)*** *^{ND4L}Glu66 (neutral) → H_2O → Glu131 (deprotonated), ND4L-ND2 interface (Fig. 5B).*

Table S1. QM regions considered in our calculations (shown in Fig. 1B of the main text). Amino acid numbers and water ids are based on PDB entry 7O71.

	Number of atoms	Residues and waters included in the QM region
L region	120-122	Lys241, Tyr316, Ser288, Tyr413, Tyr312, Tyr295, Lys383;<br>6 water molecules (ids 604, 632, 658, 679, 698, 749)
R region	160-162	Tyr225, Lys241, Ser237, Lys211, Ser165, Glu131, Ser134, Tyr138, Tyr157;<br>17 water molecules (ids 105, 604, 621, 645, 660, 675, 679, 681, 686, 691, 693, 694, 701, 703, 731, 736, 749)
"big QM region" (combined R and L region)	241-242	Tyr225, Lys241, Ser237, Lys211, Ser165, Glu131, Ser134, Tyr138, Tyr157, Tyr316, Ser288, Tyr413, Tyr312, Tyr295, Lys383;<br>20 water molecules (ids 105, 604, 621, 632, 645, 658, 660, 675, 679, 681, 686, 691, 693, 694, 698, 701, 703, 731, 736, 749)
ND4L-ND2 interface	138-139	Glu131, Lys211, Tyr138, Tyr157, Ser165, Ser134, Tyr135, ^{ND4L}Glu66, ^{ND4L}Glu30;<br>10 water molecules (ids 102, 105, 115, 117, 660, 675, 693, 694, 703, 701)
ND2-ND4 interface	87	Lys383, ^{ND4}Tyr138, ^{ND4}Ser175, ^{ND4}Glu142, ^{ND4}Lys221;<br>9 water molecules (ids 634, 658, 687, 697, 708, 719, 722, 728, 731)
Tyr157-Tyr225 benchmark	34	Tyr157, Tyr225;<br>1 water molecule (id 621)
Glu66-Glu131 benchmark	24	Glu66, Glu131;<br>1 water molecule (id 102)

Table S2. Simulated systems for the L region

**Setup #**	**Presence of $H3O^+$ (water id, Fig. S1A)**	**Charge states of the residues**		**Total charge of the QM region**	**Charge state of E131 (MM region)**	**Unbiased trajectory**	**Umbrella sampling**
		**K241**	**K383**				
**1**	-	+1	0	**+1**	-1	5.5 ps	Y413 – K383 (25 windows); K241-K383 (64 windows)
**1_1**	(W698)			**+2**		1.0 ps	-
**1_2**	(W604)			**+2**		1.0 ps	-
**1_3**	(W679)			**+2**		1.0 ps	Y413 – K383 (26 windows)
**2**	-	+1	0	**+1**	0	3.0 ps	Y413 – K383 (25 windows)
**3**	-	0	0	**0**	-1	1.5 ps	-
**3_1**	(W604)			**+1**	-1	1.5 ps	-
**4**	-	+1	+1	**+2**	0	3.0 ps	-
**5**	-	+1	+1	**+2**	-1	3.0 ps	-

Table S3. QM/MM MD systems for the R region

**Setup #**	**Presence of H3O$^+$ (water id, Fig. S1B)**	**Charge states of the residues**					**Total charge of the QM region**	**Unbiased trajectory**	**Umbrella sampling**
		**E131**	**K211**	**Y157**	**Y225**	**K241**			
**1**	-						**0**	2.0 ps	Y225 – K241 (15 windows)
**1_1**	(W621)	-1	+1	0	0	0	**+1**	1.0 ps	-
**1_2**	(W693)						**+1**	1.0 ps	-
**2**	-	-1	+1	0	-1	0	**-1**	1.0 ps	Y157 – Y225 (16 windows)
**3**	-	0	+1	-1	0	+1	**+1**	2.0 ps	K211 – Y157 (23 windows)
**4**	-						**+1**	2.0 ps	Y225 – K241 (16 windows)
**4_1**	(W693)	0	+1	0	0	0	**+2**	0.4 ps	-
**4_2**	(W701)						**+2**	1.0 ps	-
**5**	-	0	+1	0	0	+1	**+2**	1.0 ps	-
**6**	-	0 (proton on OE1)	+1	0	0	0	**+1**	1.0 ps	-
**7**	-	-1	+1	-1	0	+1	**0**	1.0 ps	-
**8**	-	0	+1	0	-1	0	**0**	1.0 ps	Y157 – Y225 (20 windows); K211-Y225 (43 windows)
**9**	-	0	+1	0	-1	+1	**+1**	1.0 ps	Y157-Y225 (17 windows)

Table S4. Simulated systems for the combined "big QM region": the combination of R and L regions into one QM region.

**Setup #**	**Charge states of the residues**			**Total charge of the QM region**	**Unbiased trajectory**	**Umbrella sampling**
	**K241**	**K383**	**E131**			
**1**	+1	0	-1	0	3.5 ps	-
**2**	+1	0	0	+1	3.5 ps	-

Table S5. Simulated systems for the "ND2-ND4" interface

**Setup #**	**Charge states of the residues**			**Total charge of the QM region**	**Unbiased trajectory**	**Umbrella sampling**
	**K383**	^{ND4}**E142**	^{ND4}**K221**			
**1**	+1	-1	0	0	2 ps	K383-^{ND4}E142 (30 windows)

Table S6. Simulated systems for the "ND4L-ND2" interface

**Setup #**	**Charge states of the residues**				**Total charge of the QM region**	**Unbiased trajectory**	**Umbrella sampling**
	^{ND4L}**E30**	^{ND4L}**E66**	**E131**	**K211**			
**1**	0	0	-1	+1	0	1 ps	-
**2**	0	0	-1	0	-1	1 ps	^{ND4L}E66-E131 (17 windows)
**3**	0 (proton on OE1)	0	-1	0	-1	2 ps	^{ND4L}E66-E131 (17 windows); ^{ND4L}E30-E131 (33 windows)

Movie S1. Cooperative nature of the proton transfer in the L region (Table S2, setup 1). Proton transfer from Tyr413 to Lys383 (shown in orange) is biased, resulting in spontaneous reprotonation of Tyr413 from Lys241 (blue arrow shows direction of proton transfer).

Movie S2. A simulated trajectory for the proton pathway Lys241 (protonated)→H2O→Tyr316 (neutral)→H2O→Ser288 (neutral)→Tyr413 (neutral)→H2O→H2O→Lys383 (neutral) in the L-region. The corresponding free energy profile is shown in Fig. 2 of the main text. The timestamp 15-30 s ("deprotonated" Tyr413/Ser288 region) corresponds to energy maximum in range -3 to -2 Å in the free energy profile. The timestamp 42 - 60 s ("deprotonated" Tyr316) corresponds to the energy maximum in range 1 -2 Å on abscissa. The movie is created by concatenating the trajectories of 50 umbrella sampling windows with the reaction coordinate values -5.3...5.7 A (taking the last 1 ps from each trajectory).

Movie S3. Cooperative nature of the proton transfer in the ND4L-ND2 (Table S6, setup 3). Proton transfer from ^{ND4L}Glu66 to Glu131 (shown in orange) is biased, resulting in spontaneous reprotonation of Glu66 from ^{ND4L}Glu30 (blue arrow shows direction of proton transfer).

References

1. *Saito, Keisuke et al. "Energetics of proton release on the first oxidation step in the water-oxidizing enzyme." Nature communications 6.1 (2015): 8488.*

2. *Katoh, Kazutaka, et al. "MAFFT: a novel method for rapid multiple sequence alignment based on fast Fourier transform." Nucleic acids research 30.14 (2002): 3059-3066.*

3. *Waterhouse, Andrew M., et al. "Jalview Version 2—a multiple sequence alignment editor and analysis workbench." Bioinformatics 25.9 (2009): 1189-1191.*
